# Liquid fermented cereals with added *Pediococcus acidilactici* did not reduce post-weaning diarrhea in pigs – an *Escherichia coli* challenge study

**DOI:** 10.3389/fvets.2023.1147165

**Published:** 2023-05-12

**Authors:** Jiajia Xu, Samantha Joan Noel, Charlotte Lauridsen, Helle Nygaard Lærke, Nuria Canibe

**Affiliations:** Department of Animal and Veterinary Sciences, Aarhus University, Tjele, Denmark

**Keywords:** post-weaning diarrhea, fermented liquid feed, *Pediococcus acidilactici*, feeding strategy, *Escherichia coli*

## Abstract

The effect of feeding fermented liquid feed (FLF) with added *Pediococcus acidilactici* to weaning piglets challenged with enterotoxigenic *Escherichia coli* (ETEC) F4 on aspects of diarrhea, performance, immune responses, and intestinal epithelial barrier function was investigated. A total of 46 weaners (weaning at 27–30 days of age) were assigned to four treatments: (1) Non-challenged and dry feed (Non-Dry); (2) Challenged and dry feed (Ch-Dry); (3) Non-challenged and FLF (Non-Ferm); (4) Challenged and FLF (Ch-Ferm). All groups received the same feed, either dry (Non-Dry and Ch-Dry), or in liquid form (Non-Ferm and Ch-Ferm) in which the cereals with added *P. acidilactici* (10^6^ CFU/g cereals) had been fermented for 24 h at 30°C. On day 1 and 2 post weaning, Ch-Dry and Ch-Ferm were orally inoculated with 5 mL × 10^9^ CFU ETEC F4/mL, whereas the Non-Dry and Non-Ferm received the same amount of saline. Fecal samples and blood samples were collected through the study period. The microbial composition, concentration of microbial metabolites and nutrient composition indicated that the quality of the FLF was high. In the first week, ADFI of both non-challenged groups was significantly higher (*p* < 0.05) than that of the Ch-Ferm group. The two challenged groups had higher fecal levels of *FaeG* gene (ETEC F4 fimbriae) from day 2 to 6 post weaning (*p* < 0.01), and higher risk of having ETEC F4 present in feces from day 3 to 5 post weaning (*p* < 0.05) compared to non-challenged groups, indicating the validity of the ETEC challenge model. Generally, ADG of the two groups fed FLF were numerically higher than those fed dry feed. Neither challenge nor FLF affected diarrhea. No significant differences were measured between Ch-Ferm and Ch-Dry regarding the level of plasma haptoglobin and C-reactive protein, hematological parameters or parameters related to epithelial barrier. The data indicated a low level of infection caused by the ETEC challenge, while recovery from weaning stress could be observed. The study showed that a strategy like this can be a way of providing a high level of probiotics to pigs by allowing their proliferation during fermentation.

## 1. Introduction

Post-weaning diarrhea (PWD) is an enteric disease causing considerable economic losses in pig production worldwide and leading to extensive usage of antibiotics and zinc oxide for prophylactic and therapeutic purposes. During acute outbreaks, mortality associated with PWD may reach 20–30% over a 1- to 2-month time span in infected pigs (1). Among several risk factors, enterotoxigenic *Escherichia coli* (ETEC) is an important cause of severe watery diarrhea (2). ETEC colonizes through attachment to the small intestinal epithelium before initiating an infection by secreting toxins (3, 4).

The use of liquid feed as a feeding strategy in pig nutrition is widespread (5). Fermentation of liquid feed results in a product with high numbers of lactic acid bacteria, a high concentration of lactic acid, and low pH (6). These parameters can have an antibacterial activity. It has been shown that feeding piglets with fermented liquid feed (FLF) results in a reduction in the number of Enterobacteriaceae, which include coliforms and *Salmonella*, in the gastrointestinal tract of pigs (7–9). Additionally, studies have shown beneficial effects of FLF in animals infected with *Lawsonia intracellularis* (10) and *Brachyspira hyodysenteriae* (11). However, the fermentation process of the whole feed mixture can result in the degradation of amino acids, mainly lysine, which are decarboxylated, resulting in increased concentration of biogenic amines in the feed (12, 13). The negative impact of these reactions can be three-folded, firstly, a degradation of essential amino acids will impact the nutritional value of the feed with a risk of undersupplying the animals; secondly, a high concentration of biogenic amines is considered to contribute to impaired palatability leading to reduced feed intake (14); thirdly, the presence of high concentrations of biogenic amines might have a negative health impact (15, 16). Therefore, fermenting only the cereals as opposed to the complete feed can be an alternative (17).

One of the expected advantages of feeding FLF to piglets around weaning is that it contributes to a less stressful weaning transition due to its liquid characteristics being closer to the consistency of sow’s milk. This might help avoid a drastic reduction in feed intake at weaning (8). Furthermore, adding a specific bacterial strain shown to exhibit probiotic effects, able to proliferate in the liquid feed during fermentation could lead to a better microbiological quality of the feed, as well as be a way of providing a high number of bacteria with health promoting properties (18), with the expected beneficial impact on the animals. Our previous study investigated various candidate bacteria species to be added to liquid feed by a series of *in vitro* fermentation experiments (19). A mixture of cereal grains and water (1:2.75, w:w) was prepared and one of four candidate strains added (10^7^ CFU/g liquid mixture) and incubated for 48 h at 30°C. *Pediococcus acidilactici* showed the best characteristics as a whole, i.e., lowest pH, suppressed the growth of Enterobacteria to below detection limits, and high lactic acid concentration (19). However, the impact of FLF with added *P. acidilactici* on PWD still needs to be further investigated.

The objective of this study was to investigate whether feeding piglets liquid feed based on liquid fermented cereals with added *P. acidilactici* could reduce diarrhea, improve immune responses, and intestinal epithelial barrier function, and increase performance during the post-weaning period. For that, an *in vivo* challenge study with an ETEC (O149, F4ac+, LT+, STb+) was conducted with piglets.

## 2. Materials and methods

### 2.1. Diets, experimental groups and feeding

**Figure 1:**
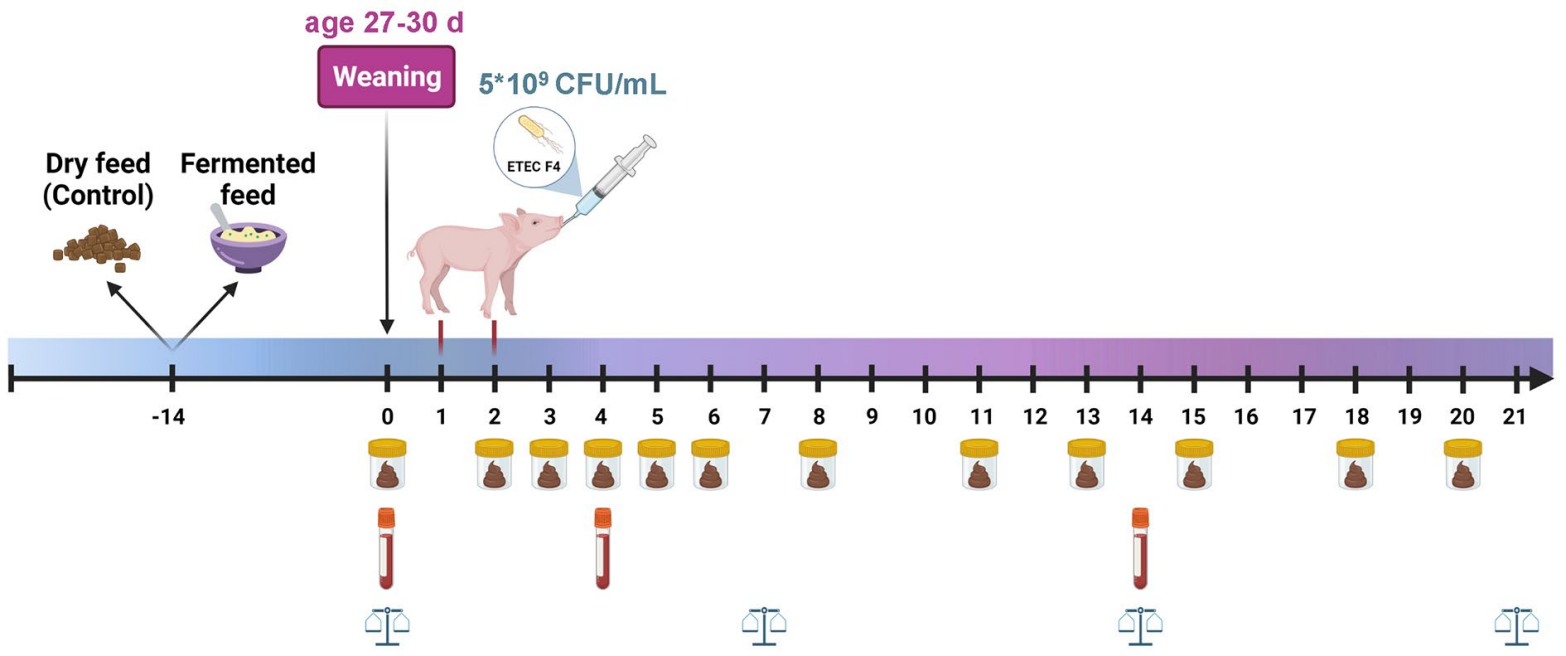
Experimental timeline. From 14 days of age, half of the litters were offered dry feed, and the other half received FLF based on the same diet. Piglets weaned at 27–30 days of age. On day 1 and 2 post weaning, the two challenge groups were inoculated with 5 mL of 10^9^ CFU ETEC/mL, the two non-challenged groups received the same amount of saline. Individual body weight was registered on weaning day (day 0), and on day 7, 14, and 21 after weaning. Fecal samples were collected day 0, 2–6, 8, 11, 13, 15, 18, and 20 after weaning. Blood samples were taken by puncture of the jugular vein on day 0, 4 and 14 post weaning. Created with BioRender.com.

Piglets were assigned to one of four experimental groups where they were challenged with ETEC or not challenged and fed either dry or fermented liquid diets based on standard Danish starter and weaner diets (Supplementary Table S1): (1) Non-challenged and dry feed (Non-Dry); (2) Challenged and dry feed (Ch-Dry); (3) Non-challenged and fermented liquid feed (Non-Ferm); (4) Challenged and fermented liquid feed (Ch-Ferm; Figure 1). The Dry groups (Non-Dry and Ch-Dry) received the diets in dry form, and the Ferm groups (Non-Ferm and Ch-Ferm) received the same diets in liquid form in which the cereals with added *P. acidilactici* had been fermented (see below 2.2 for further description).

The ETEC strain *E. coli* F4 O149 (Aarhus University), which has the genes for and produces heat-stable enterotoxin b (STb), heat-labile toxin (LT) and adhesin F4ac was used to challenge the piglets. To prepare the strain for inoculation, ETEC F4 was aerobically grown overnight on blood agar plates at 37°C (Columbia sheep blood agar, 100253ZFMP, VWR International BVBA, Leuven, Belgium), a single colony was transferred to BHI broth (1.10493, Merck KGaA, Darmstadt, Germany) and incubated at 37°C for 5 h (150 rpm constant shaking). Cells were harvested by centrifugation at 15938 g for 10 min, the pellet was diluted with 0.9% NaCl until OD_600_ reached 1.0, which corresponded to ~10^9^ colony-forming unit (CFU)/mL. Colony forming units (CFU) of the suspension were determined on blood agar plates after overnight aerobic incubation at 37°C to verify the number of bacteria in the suspension. On day 1 and 2 post weaning, the challenged groups (Ch-Dry and Ch-Ferm) were orally inoculated with 5 mL of the solution containing 10^9^ CFU ETEC/mL by using a syringe with a soft rubber mouthpiece. The non-challenged groups (Non-Dry and Non-Ferm) received the same amount of sterile saline.

Until day 13 of age, all piglets exclusively received milk from the sow. From 14 days of age until 7 days after weaning (weaning at 27–30 days of age), half of the litters were offered dry feed based on the starter diet, and the other half received FLF based on the same starter diet (Supplementary Table S1). From 7 days until 21 days after weaning, the litters continued the same dry or FLF diets but based on the weaner diet (Supplementary Table S1). Piglets had *ad libitum* access to feed and water throughout the experiment. Sows were fed a standard Danish sow diet and with amounts according to Danish norms (20).

### 2.2. Production of fermented liquid feed

The fermented cereals were prepared by mixing the cereal portion of the diets (69.8% wheat and 30.2% barley) with water at the ratio 1:2.4 (w:w) and 10^6^ CFU *Pediococcus acidilactici* (Bactocell, CNCM MA18/5 M)/g cereals were added. The mixture was then incubated for 24 h at 30°C, with 1 min of stirring every hour. After 24 h, the fermented cereals were transferred to a mixing tank to be mixed with the remaining ingredients of the diet, as well as water, aiming at a final dry matter (DM) content of approximately 26%. The complete feed mixture was stored at 20°C to reduce the risk of further fermentation and thus maintain its good quality. Benzoic acid 0.5% was added in the compound feed also to minimize the further fermentation process. Before feeding, the complete feed mixture was first stirred in the mixing tank for 50 s, then conveyed to the pipes mounted indoors for circulation, while the mixing tank stirred the circulating feed, this process lasted 1 to 2 min to ensure the homogeneity of the feed. The feed mixture was automatically pneumatic conveyed and fed to the piglets using a liquid feeding system (Bopil A/S, Sønderborg, Denmark) that was set to feed every hour if the trough was detected as almost empty. Two sets of tanks were used to produce the experimental feed; when the feeding from one set began, the cereal mixture was prepared in the other set, so that a new batch of fermented cereals with *P. acidilactici* was ready 24 h later, then mixed with the remaining ingredients and fed. To ensure no contamination, the troughs were emptied every day, and the tanks were set to self-clean every 24 h.

### 2.3. Animals

The study was performed at Aarhus University, Department of Animal and Veterinary Sciences (AU Viborg, Denmark). The animal and experimental procedures were approved by the Danish Animal Experiments Inspectorate, Ministry of Food, Agriculture and Fisheries, Danish Veterinary and Food Administration. Animal care and housing were in accordance with Danish laws and regulations governing the humane care and use of animals in research (LBK no. 474 of 15/05/2014). A total of 46 piglets from 15 sows (Duroc × Landrace × Yorkshire mated with Norsvin Landrace boar, first parity) were used in this study. The sows were confirmed to be homozygous carriers of the dominant gene (*MUC4* gene) coding for intestinal ETEC F4 fimbriae receptors using competitive allele specific PCR (KASP) (VHL Genetics, Netherlands); thus, piglets were genetically susceptible to ETEC F4 (21). The study was carried out in two blocks. Eight litters were included in the first block and seven in the second (one of the initial eight sows farrowed too few piglets and therefore could not be included in the study). Starting from day 14 of age, piglets were fed with either dry or FLF diets. At the beginning of the feeding trial, the body weight of piglets offered dry diet was 4.14 ± 0.58 kg, and the body weight of piglets offered FLF diet was 4.15 ± 0.69 kg. At weaning, two littermates from each sow of the two dietary groups were allocated to the two challenged groups, i.e., Ch-Dry (*N* = 14 from 7 sows) and Ch-FLF (*N* = 16 from 8 sows), respectively. From four of the sows, two littermates were allocated to the two non-challenged groups, i.e., Non-Dry (*N* = 8 from 4 sows) and Non-FLF (*N* = 8 from 4 sows). Piglets were distributed in the experimental groups according to weight to reach a similar average body weight across groups.

### 2.4. Housing, registrations and sampling

New-born piglets were housed in pens for loose-housed sows (Type FT30, 3.0 × 2.2 m, Skiold Jyden A/S, DK) until weaning. We provided new-born piglets with additional heat using a 150 W bulb in the corner for the first 7–10 days until they could maintain sufficient warmth solely with the floor heating. After weaning, pairs of littermates were housed in the same pen (215 cm × 110 cm), with 75 cm × 110 cm slatted floor, and 140 cm × 110 cm concrete floor with floor heating and partial coverage. All piglets were housed in the same room, with piglets receiving dry feed and those receiving FLF placed on either side of the aisle. Furthermore, an empty pen was allowed between the challenged and non-challenged groups to prevent cross-contamination. No physical contact was allowed between piglets from different pens. Shoe covers and disposable protective suits were worn when the personnel entered the room; gloves, plastic sleeves, aprons, and boots were changed between handling non-challenged groups and challenged groups. Non-challenged pigs were always handled before ETEC-challenged pigs. To avoid affecting the gastrointestinal system and experimental parameters, no bedding was allowed. Each pen was provided with a rope, which could help to satisfy the natural behavior of the piglets. The room temperature was 25°C the first week after weaning and then gradually reduced to 21°C the third week. The humidity in the room was 62.1%, and the ventilation rate was 3.85 cfm/pig in both blocks.

Individual body weight was registered on the day of weaning (day 0), and on day 7, 14, and 21 after weaning (Figure 1). Feed intake per pen was registered daily and was calculated on a DM feed intake basis. Fecal samples were collected directly from the rectum on weaning day (day 0), and on days 2–6, 8, 11, 13, 15, 18, and 20 after weaning, using a plastic glove lubricated in gel. The individual fecal samples were immediately scored according to consistency following a 7-score scale which consisted of the following categories: (1) hard, dry and lumpy; (2) firm; (3) soft yet malleable; (4) soft and liquid; (5) watery and dark; (6) watery and yellow; (7) foamy and yellow. A score > 3 was considered as diarrhea. The samples were divided into three aliquots for DNA extraction followed by quantitative real-time polymerase chain reaction (qPCR) (stored at −80°C), DM determination (stored at −20°C), and microbiological enumeration (conducted immediately). Blood samples were taken by puncture of the jugular vein on day 0 (weaning day), day 4 and day 14 post weaning. Blood samples were drawn into EDTA containing-vacutainers for hematology analysis, and heparinized tubes for analysis of cytokine, C-reactive protein (CRP), haptoglobin, diamine oxidase activity (DAO) and lipopolysaccharide (LPS) concentration. Plasma was separated by centrifugation at 1300 g and 4°C for 10 min and then stored at −80°C until analysis.

### 2.5. Analytical methods

#### 2.5.1. Chemical analysis

Dry matter content of feces and dry feed was determined by freeze drying. The DM of the FLF was determined by drying to constant weight at 103°C (≈20 h). Ash was measured according to method 923.03 AOAC (22). Crude protein (N × 6.25) was determined by the Dumas method (23) and amino acids were determined using the European Economic Community methods (24). Crude fat was determined by HCl-Bligh and Dyer extraction as described by Jensen (25). Gross energy was measured on a 6,300 Automatic Isoperibol Calorimeter system (Parr Instruments, Moline, IL, United States). The dietary content of starch, soluble and insoluble non-starch polysaccharides (NSP), non-cellulosic polysaccharides (NCP), cellulose, and Klason lignin were analyzed essentially as described by Bach Knudsen except that acid hydrolysis was performed in 2 M-H_2_SO_4_ for 1 h instead of 1 M-H_2_SO_4_ for 2 h (26). Digestible sugars and non-digestible oligosaccharides (NDO) were determined after extraction in 50% ethanol by HPLC-PAD (27). Biogenic amine (cadaverine, agmatine, putrescine and tyramine) concentration was determined using HPLC as described by Poulsen (28). Organic acid concentration were determined as described by Canibe et al. (12).

#### 2.5.2. Microbiological enumeration

To enumerate selected microbial groups in the FLF, samples (approximately 5–10 g) were transferred to blender bags (400 mL, No.129–0729, VWR International BVBA, Leuven, Belgium) containing a peptone solution in a 10-fold dilution and homogenized for 2 min in a smasher paddle blender (bioMérieux Industry, United States). After homogenization, serial 10-fold dilutions were prepared in a peptone solution and 100-μL aliquots were spread-plated on agar plates (except 1 mL pour-plated on TSC agar plates). Enterobacteriaceae were enumerated on MacConkey agar (84614.0500, VWR International BVBA, Leuven, Belgium) after overnight aerobic incubation at 37°C. Yeasts and mold were enumerated on YM agar (85052.0500, VWR International BVBA, Leuven, Belgium) with Chlortetracycline and Chloramphenicol supplement (SR0177E, Oxoid LTD, Basingstoke, England) after aerobic incubation for 3 days at 30°C. *Clostridium perfringens* were enumerated using the pour-plate technique on TSC agar (84636.0500, VWR International BVBA, Leuven, Belgium) after anaerobic incubation for 3 days at 30°C. Lactic acid bacteria were enumerated on MRS agar (84607.0500, VWR International BVBA, Leuven, Belgium) after anaerobic incubation for 3 days at 30°C. Bacteria and yeasts were counted using a manual colony counter and the result expressed as CFU/g feed. Detection limits for microorganisms were as 3 log CFU/g sample for Lactic acid bacteria, Enterobacteriaceae and yeasts, 2 log CFU/g sample for *Cl. perfringens*.

To determine ETEC counts in feces, 1–3 grams of fecal sample was suspended in a peptone solution (1:10, w:v) and homogenized for 2 min in a smasher paddle blender (bioMérieux Industry, United States). After homogenization, serial 10-fold dilutions were prepared in peptone solution and 100-μL aliquots were spread on to blood agar plates (100253ZFMP, VWR International BVBA, Leuven, Belgium) and incubated aerobically overnight at 37°C. Hemolytic bacteria were counted using a manual colony counter and the result expressed as CFU/g feces. Blood agar plates with hemolytic colonies were stored at 5°C until ETEC F4 serotyping was performed using the slide agglutination test with type-specific antisera (SSI Diagnostica A/S, Hillerød, Denmark) on three randomly selected colonies per plate. The limit of detection was 5 log CFU/g feces.

#### 2.5.3. Quantification of fecal F4ac fimbriae, LT, STb, and *Pediococcus acidilactici*

DNA was extracted from 50 mg feces samples using NucleoSpin 96 DNA Stool kit (MACHEREY-NAGEL, Germany) following the manufacturer’s protocol, with modifications. After the addition of Lysis Buffer ST1, samples in MN Bead Tube were mechanically homogenized using a homogenizer (star-beater, VWR International BVBA, Leuven, Belgium) at a frequency of 30 Hz for 5 min. DNA was eluted in 150 μL of elution buffer and stored at −20°C until further analysis. DNA concentration was determined using a Qubit Fluorometer.

Quantitative real-time PCR (qPCR) was performed on an Applied Biosystems ViiA7 real-time PCR system (Thermo Fisher Scientific, United States) using MicroAmp Optical 384-well reaction plate (Applied Biosystems). The reaction system contained 5 μL of RealQ Plus 2x Master Mix Green (Amplicon, Denmark), 0.3 μL forward and reverse primers, 2 μL of DNA template, and nuclease-free water which was used to adjust the final volume to 10 μL per well. The primers were purchased from Sigma-Aldrich (Darmstadt, Germany), primer sequences are listed in Table 1.

**Table 1:** Sequences of primers used for real-time PCR.

Target Gene	Primer direction^#^	Sequence	Reference
*FaeG* (F4ac fimbriae)	F	CACTGGCAATTGCTGCATCT	(29)
	R	ACCACCGATATCGACCGAAC	
*eltB* (LT toxin)	F	AAGCCATTGAAAGGATGAAGGA	(29)
	R	CTGATTGCCGCAATTGAATT	
*est-II* (STb toxin)	F	TGCCTATGCATCTACACAAT	(30)
	R	CTCCAGCAGTACCATCTCTA	
*P. acidilactici* (*D-LDH*)	F	GGACTTGATAACGTACCCGC	(31)
	R	GTTCCGTCTTGCATTTGACC	

# F, forward; R, reverse.

Determination for DNA samples, standards, and non-template control were conducted in triplicates on each plate. The temperature profile was as follows: initial heating at 50°C for 2 min, followed by hot start activation at 95°C for 15 min and 40 cycles of amplification. For the ETEC F4 fimbriae (*FaeG* gene), LT toxin (*eltB* gene) and *P. acidilactici* (*D-LDH* gene), annealing temperature was 60°C for 30 s and extension 72°C for 30 s. For the STb toxin (*est-II* gene), annealing and extension were 59.1°C for 60 s. Melting curve analysis was done by increasing the temperature to 95°C at a rate of 0.05°C/s with recording continuously. Target gene copy numbers in the original sample were determined from a standard curve consisting of a 5-fold serially diluted DNA standards with a known copy number. *Enterotoxigenic E. coli* F4 and *P. acidilactici* pure cultures were used as standards. Target gene copy number was determined in the standards using DNA copy number and dilution calculator (32).

The Ct cut-off values were 29 for F4 fimbriae (*FaeG* gene), 27 for LT toxin (*eltB* gene), 30 for STb toxin (*est-II* gene) and *P. acidilactici* (*D-LDH* gene).

#### 2.5.4. Hematology and immune parameters

Complete blood count was performed as haematology diagnostics using ProCyte Dx Haematology Analyser (IDEXX, United States). Blood plasma haptoglobin and CRP concentrations were measured using Phase range Haptoglobin Assay Kit (cat. no. TP-801) and Phase porcine CRP Assay Kit (cat. no. TA-901) respectively (Tridelta Development Ltd., Ireland). Haptoglobin analyses were performed using an ADVIA 1800 Clinical Chemistry System autoanalyzer (Siemens Healthineers AG, Germany), while CRP concentrations were determined by solid-phase sandwich immunoassay, and measured using a POLARstar Galaxy microplate reader (BMG Labtech, Germany). The plasma concentration of cytokines (IFN-γ, IL-1β, IL-6, IL-10, IL-12, and TNF-α) were analyzed using methods previously described (33). Plasma diamine oxidase activity (DAO) was determined by a kinetic-fluorometric method where 1.5 diamino pentane (cadaverine, Sigma C8561) was the substrate and 10-acetyl-3,7-dihydroxyphenoxazine (ADHP) was the profluophore oxidized by the developed hydrogen peroxide. Units were defined as d-emission per min at 590 nm after excitation at 544 nm. Plasma lipopolysaccharide (LPS) concentration was analysed using the Porcine Endotoxin ELISA kit (AMSBIO, United Kingdom). The assays were performed in duplicates.

### 2.6. Calculations and statistical analysis

Feed intake of the pigs fed with FLF was calculated by daily weighing the feed offered and the leftovers and determining the DM content of the feed leftovers weekly. The qPCR data were analyzed by setting a limit of quantification (LOQ) log copies first, i.e., 5 gene copies per 2 μL DNA elution, then LOQ log copies/g sample was calculated according to the exact weight of the sample used for DNA extraction.

Statistical analyses were performed using R Studio (version 4.2.1) (34). QQ plots were used to assess if the assumption of model residuals being normally distributed was met. Model diagnostics were conducted in the R package “DHARMa” (35) and “performance” (36). For all analyses, the emmeans package was used to compare between treatment groups. Significance was declared at *p* ≤ 0.05, and values 0.05 < *p* < 0.10 were considered a tendency. *p*-values from pair-wise comparison were adjusted using the Bonferroni adjustment.

Treatment group, day and their interaction were included as fixed effects in all analyses. Pen, sow, block, and pig were included as random effects, and removed from the model if their effect was not significant except pig, or if a convergence problem was encountered.

Linear mixed-effects models were used to analyze all response parameters using lme4 package (37), except the probability of diarrhea, and fecal shedding of ETEC F4 based on plate counting. Body weight at weaning was used as a covariate for ADG and ADFI analyses. Tests for neutrophils, eosinophils, and all cytokines (except IFN-γ and IL-12) data were performed on log scale.

Individual feces score was analyzed using a binomial distribution, by which score <3 was considered as non-diarrheic samples and score >3 was considered as diarrhea. A generalized linear mixed model was used, with a logit link to convert values to the scale of a probability (38).

The data on fecal shedding of ETEC F4 based on plate counting were analyzed assuming a penalized maximum likelihood binomial distribution (Bayesian mixed-effects model), using the blme package (39), where samples were classified as below or above the detection limit, which was 5 log CFU/g feces. Due to the nature of this data, i.e., too many samples below the detection level, linear mixed-effects models could not be applied to this dataset, thus the raw data are shown in Supplementary Figure S2.

## 3. Results

### 3.1. Feed composition

The chemical and microbial composition of the experimental diets are shown in Tables 2, 3, respectively. The concentration of sugars decreased by fermentation of the cereals to approximately half of the initial concentration and a further reduction in their concentration was observed after 24 h of storage of the complete feed at 20°C, reaching values of 6–28% of the initial levels (Table 2). This decrease was also observed in the concentration of NDO, reaching values of 6–14% of the initial levels. The concentration of starch was slightly lower in the FLF samples compared to the dry feed, and a similar pattern was seen for NSP. The concentration of lysine and all other amino acids was similar in the FLF and dry feed.

**Table 2:** Chemical composition of the experiment diets (*N* = 1).

Items	Day 0–7 post weaning			Day 8–21 post weaning		
	Control dry feed	FLF-1 h^1^	FLF-24 h^2^	Control dry feed	FLF-1 h^1^	FLF-24 h^2^
**Chemical composition**						
DM (g/kg)	896.72	213.53	187.61	895.10	213.53	187.61
Ash (g/kg DM)	53.60	48.26	50.17	54.42	53.05	51.29
Crude protein (*N* × 6.25) (g/kg DM)	222.84	219.35	219.70	208.77	213.15	214.27
Fat (g/kg DM)	32.87	28.95	27.67	25.82	24.73	26.55
Energy (MJ/kg DM)	18.25	18.15	18.23	17.89	17.52	17.93
Digestible CHO (g/kg DM)^3^	524.8	498.4	500.6	551.8	504.3	484.4
Starch	507.3	488.0	495.6	532.7	497.3	483.3
Sugars^4^	17.5	10.4	4.9	19.1	7.0	1.1
Dietary fibre^5^	134.3	131.2	132.5	141.5	145.5	135.4
Non-digestible CHO^6^	117.9	109.6	108.4	124.1	111.0	108.2
NDO^7^	5.1	0.8	0.3	9.3	2.8	1.3
Total NSP (soluble NSP)	112.8 (30.8)	108.8 (48.8)	108.0 (27.5)	114.7 (33.4)	108.3 (32.9)	106.9 (24.1)
Total NCP	92.9	87.5	87.5	95.2	87.3	84.9
Cellulose	19.9	21.3	20.5	19.6	20.9	22.0
Klason lignin	16.4	21.6	24.1	17.4	34.5	27.2
**Amino acids (g/kg DM)**						
Ala	9.4	9.6	10.1	8.5	9.0	9.3
Arg	12.9	12.5	12.4	12.0	11.8	11.9
Asp	19.3	19.1	19.5	18.2	18.7	19.1
Cys	3.5	3.4	3.5	3.4	3.3	3.4
Glu	41.0	41.9	42.8	40.1	39.9	39.8
Gly	9.5	9.6	9.9	8.7	9.0	9.1
His	5.0	4.8	4.9	4.7	4.7	4.7
Ile	9.7	9.8	10.0	9.1	9.4	9.6
Leu	16.0	16.2	16.6	15.2	15.6	15.9
Lys	15.2	14.6	15.1	13.8	14.2	14.1
Met	4.7	4.5	4.6	4.2	4.2	4.1
Orn	U^8^	0.4	0.5	U	0.4	0.5
Phe	10.6	10.7	11.0	10.2	10.5	10.5
Pro	14.0	14.7	15.1	13.8	13.9	13.9
Ser	10.6	10.7	10.9	10.2	10.3	10.4
Thr	9.6	9.6	9.7	8.8	9.1	9.0
Val	11.8	12.0	12.3	10.7	11.0	11.3

1 Fermented cereals mixed with the remaining feed ingredients and samples taken after 1 h stirring.

2 Fermented cereals mixed with the remaining feed ingredients and samples taken after 24 h of storage.

3 Digestible carbohydrates (CHO) = starch + monosaccharides + disaccharides + maltotriose.

4 Sugars = monosaccharides + disaccharides + maltotriose.

5 Dietary fibre = non-digestible oligosaccharides (NDO) + non-starch polysaccharides (NSP) + Klason lignin.

6 Non-digestible CHO = NSP + oligosaccharides except maltotriose.

7 NDO = Raffinose + Stachyose + Verbaskose + 1-Kestose + Kestotetraose (Nystose).

8 “U” means under the detection limit.

**Table 3:** Microbial composition (log CFU/g sample) and concentration of organic acids (mmol/kg sample) and biogenic amines (mg/kg sample) in the liquid feed offered from day 0 to 7 post-weaning and from day 8 to 21 post-weaning^1^.

	Feed day 0–7 post weaning				Feed day 8–21 post weaning			
	Cereals-0 h^2^	Cereals-24 h^3^	FLF-1 h^4^	FLF-24 h^5^	Cereals-0 h^2^	Cereals-24 h^3^	FLF-1 h^4^	FLF-24 h^5^
**Microbial groups** ^**6**^								
Lactic acid bacteria	5.89 ± 0.57	8.80 ± 0.15	8.68 ± 0.49	8.76 ± 0.71	5.82 ± 0.32	8.61 ± 0.43	8.94 ± 0.27	9.00 ± 0.30
*P. acidilactici*	5.83 ± 0.73	8.20 ± 0.43	7.84 ± 0.88	8.06 ± 0.70	5.49 ± 0.11	7.67 ± 0.45	7.63 ± 0.37	7.90 ± 0.39
*Cl. perfringens*	2.23 ± 0.34	2.00 ± 0.00	2.18 ± 0.38	2.04 ± 0.14	2.16 ± 0.29	2.18 ± 0.35	2.34 ± 0.36	2.43 ± 0.47
Enterobacteriaceae	5.14 ± 0.17	3.28 ± 0.71	3.30 ± 0.43	3.20 ± 0.53	5.13 ± 0.40	3.07 ± 0.27	3.31 ± 0.52	3.11 ± 0.21
Yeasts	3.88 ± 0.79	6.49 ± 0.98	6.29 ± 0.93	5.77 ± 1.39	3.83 ± 0.41	5.84 ± 1.17	5.74 ± 1.03	4.79 ± 0.73
pH	6.15 ± 0.18	3.86 ± 0.13	4.77 ± 0.21	4.48 ± 0.20	6.11 ± 0.18	3.71 ± 0.11	4.67 ± 0.08	4.35 ± 0.11
**Organic acids, mmol/kg sample** ^**7**^								
Formic acid	0	0.5 ± 1.5	1.3 ± 1.5	2.9 ± 2.7	0	0.1 ± 0.2	0.9 ± 1.1	5.2 ± 5.3
Acetic acid	1.8 ± 0.6	14.1 ± 5.9	15.2 ± 3.0	23.5 ± 5.4	0.8 ± 0.1	15.7 ± 2.9	15.8 ± 1.2	23.0 ± 6.6
Benzoic acid	0	0	10.4 ± 1.0	10.4 ± 1.0	0	0	9.0 ± 0.5	7.0 ± 2.0
DL-Lactic acid	0.5 ± 1.7	67.7 ± 19.6	58.4 ± 10.6	90.8 ± 21.1	0	79.7 ± 15.1	68.8 ± 6.3	93.0 ± 27.4
Succinic acid	0.1 ± 0.3	2.4 ± 0.8	2.1 ± 0.4	2.4 ± 0.3	0	1.7 ± 0.6	1.8 ± 0.3	3.0 ± 0.9
**Biogenic amines, mg/kg sample**								
Cadaverine	0	13.5 ± 11.0	22.8 ± 8.1	22.0 ± 9.4	0	9.1 ± 7.7	12.9 ± 5.5	17.9 ± 10.3
Agmatine	32.6 ± 3.4	40.1 ± 13.1	51.6 ± 6.8	49.3 ± 4.9	30.4 ± 4.6	37.4 ± 4.3	46.3 ± 2.7	35.5 ± 10.7
Putrescine	3.3 ± 0.4	10.3 ± 6.4	15.9 ± 6.2	36.4 ± 23.7	2.9 ± 0.7	8.5 ± 4.0	11.4 ± 5.5	28.4 ± 20.0
Tyramine	0	16.3 ± 14.1	16.8 ± 11.1	22.2 ± 16.4	0	29.6 ± 8.8	24.5 ± 7.9	21.5 ± 11.5
Total	35.9 ± 3.7	80.1 ± 44.6	107.0 ± 32.2	129.8 ± 54.4	33.2 ± 5.3	84.6 ± 24.7	95.1 ± 21.7	103.3 ± 52.5

1 Values are means and standard deviation. *N* = 12–15, except *P. acidilactici* (*N* = 7–8), and biogenic amines (*N* = 6–9).

2 Cereals and *P. acidilactici* and water mixed.

3 The cereal mixture after 24 h of incubation at 30°C.

4 The complete feed 1 h after mixing and stored at 20°C.

5 The complete feed 24 h after mixing and stored at 20°C.

6 The unit is log CFU/g sample, except for *P. acidilactici* is log copies/g sample. Detection levels for microorganisms are 3.0 log CFU/g sample for lactic acid bacteria, Enterobacteriaceae and yeasts; 3.8 log copies/g sample for *P. acidilactici*; and 2.0 log CFU/g sample for *Cl. perfringens*.

7 Propionic acid, Isobutyric acid, n-Butyric acid, Iso-valeric acid and n-Valeric acid under detection level.

Lactic acid bacteria counts in all fermented samples (both the cereals and the complete feed) reached values between 8.6 and 9.0 log CFU/g (Table 3). *Pediococcus acidilactici* in the fermented cereal mix reached around 8 log copies/g after 24 h of fermentation, which was more than 100 times the initial added dose. The number of Enterobacteriacea was reduced by fermenting the cereals to levels close to the detection limit, i.e., 3 log CFU/g, and kept at this level after mixing with the remaining feed ingredients. *Cl. perfringens* counts were close to detection levels (2 log CFU/g) in all samples, and mold numbers were below detection level (3 log CFU/g) in all samples (data not shown).

The pH decreased to values below 4 after fermenting the cereals for 24 h, the levels increased after mixing the remaining feed ingredients and were below 4.5 after 24 h of storage of the complete feed. Lactic acid concentration increased after fermenting the cereals for 24 h, the levels slightly decreased after mixing the remaining feed ingredients and increased again to higher levels after 24 h of storage. The concentration of biogenic amines increased by fermenting the cereals for 24 h, and a further increase was observed after 24 h of storage of the complete feed. Agmatine was measured at the greatest concentration, also in the initial cereal mixture.

### 3.2. Growth performance

No significant difference in body weight at weaning was detected between treatment groups (*p* = 0.46) (Table 4). Generally, ADG of the two groups fed FLF were numerically higher than those fed dry feed but not statistically significant. The first week post weaning, the Ch-Dry group lost weight, and almost no weight gain was observed in the Ch-Ferm group, whereas the non-challenged groups had a small positive ADG. In the first week, ADFI of both non-challenged groups was significantly higher (*p* < 0.05) than that of the Ch-Ferm group. Gain to feed ratio (G:F) during the whole experimental period was similar in all four treatment groups.

**Table 4:** Effect of ETEC F4 challenge and fermented liquid feed on body weight, average daily gain, average daily feed intake, and gain to feed ratio (G:F)^1^.

Item	Treatment^2^				SEM^3^	*p*-value
	Non-Dry	Ch-Dry	Non-Ferm	Ch-Ferm		
**Body weight (kg)**						
Day 0^4^	6.93	7.85	7.74	7.36	0.42	0.46
Day 7	7.31	7.26	7.93	7.39	0.45	0.75
Day 14	8.47	8.73	9.14	8.81	0.52	0.89
Day 21	10.60	11.30	11.90	11.80	0.77	0.68
**Average daily gain (g)**						
Day 0–7	24.1	−65.2	39.3	1.8	40.6	0.27
Day 7–14	175.0	206.0	180.0	201.0	33.7	0.86
Day 14–21	334.0	359.0	386.0	431.0	56.2	0.41
Day 0–21	178.0	167.0	198.0	210.0	33.5	0.59
**Average daily feed intake (g)**						
Day 0–7	114.2^a^	89.2^ab^	109.1^a^	66.0^b^	21.9	< 0.01
Day 7–14	243.0	237.0	209.0	206.0	19.2	0.62
Day 14–21	390.0	370.0	389.0	433.0	44.4	0.42
Day 0–21	247.0	232.0	235.0	235.0	22.6	0.77
**Gain: feed ratio**						
Day 0–7	−0.21	−0.84	0.13	−0.28	0.73	0.76
Day 7–14	0.69	0.84	0.83	0.97	0.11	0.38
Day 14–21	0.87	0.95	0.94	1.03	0.11	0.77
Day 0–21	0.72	0.70	0.79	0.92	0.11	0.37

1 Values are presented as emmeans.

2 Non-Dry: non-challenged, standard dry feed, *n* = 8; Ch-Dry: challenged, standard dry feed, *n* = 14; Non-Ferm: non-challenged, fermented liquid feed, *n* = 8; Ch-Ferm: challenged, fermented liquid feed, *n* = 16. The ETEC F4 was orally administered on days 1 and 2 post weaning.

3 Pooled standard error of least square means.

4 Day 0: the day of weaning.

ab Values within a row without a common superscript differ (*p* < 0.05).

### 3.3. Fecal consistency

**Figure 2:**
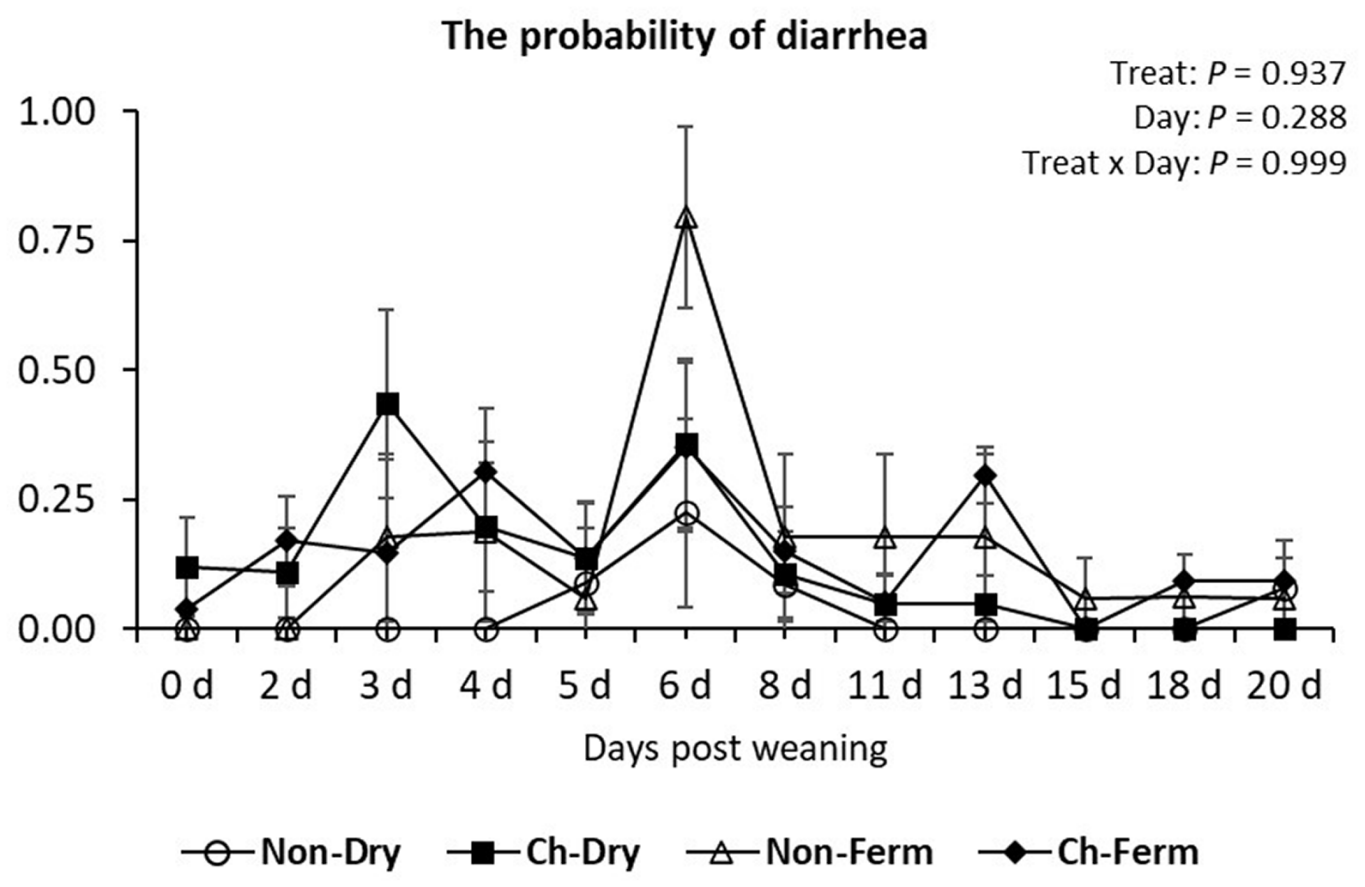
The probability of developing diarrhea (fecal score > 3) in the treatment groups. The ETEC F4 was orally administered on days 1 and 2 post weaning. Data are presented as emmean ± SEM. Non-Dry, non-challenged, standard dry feed, *n* = 8; Ch-Dry, challenged, standard dry feed, *n* = 14; Non-Ferm, non-challenged, fermented liquid feed, *n* = 8; Ch-Ferm, challenged, fermented liquid feed, *n* = 16.

There was no significant difference between treatment groups in the probability of developing diarrhea (*p* = 0.94) (Figure 2). The probability levels were below 61% at all times in all groups except the Non-Ferm group, which showed a probability of 79.6% (±17.5%) on day 6.

**Figure 3:**
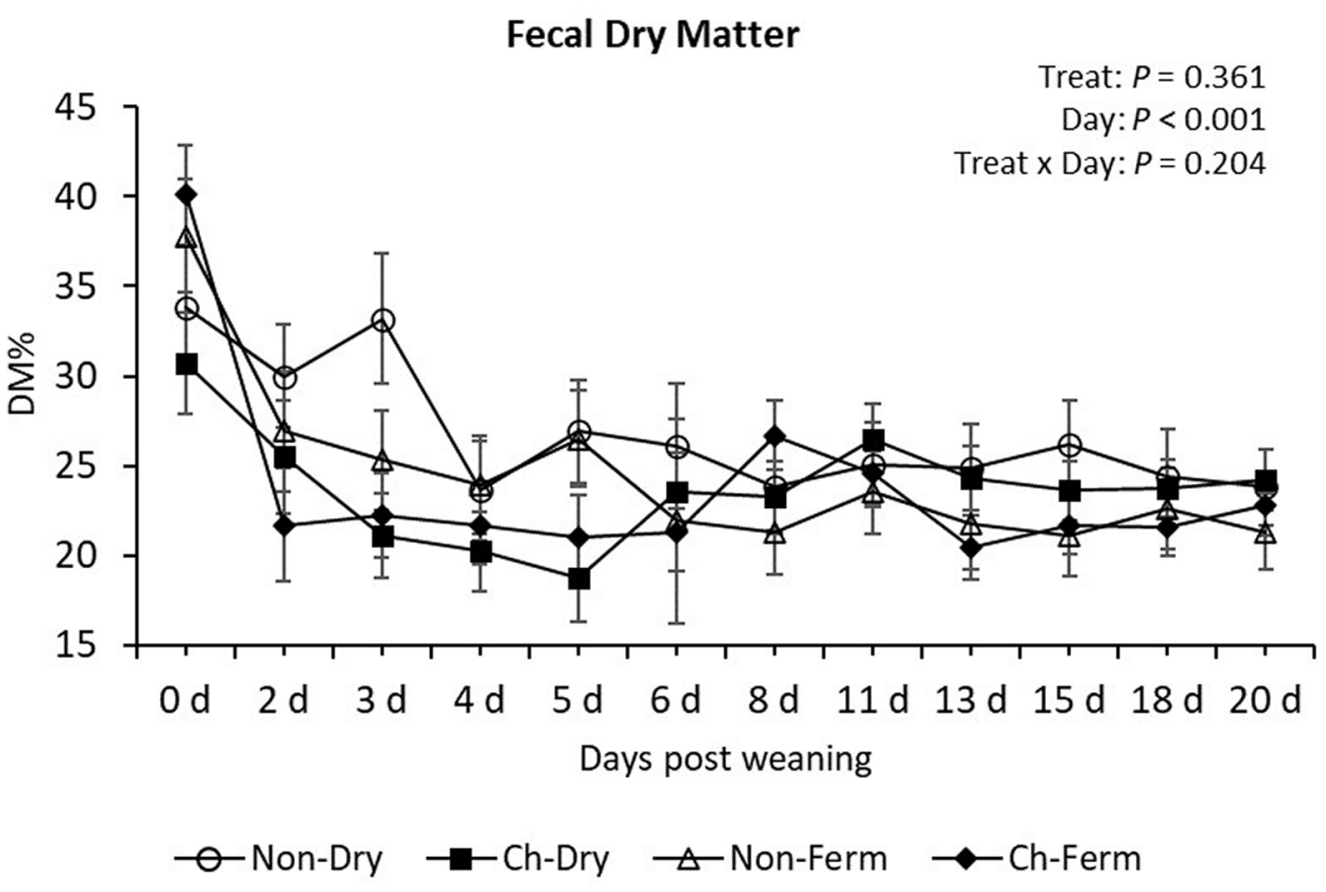
Fecal dry matter content (DM%) in treatment groups. The ETEC F4 was orally administered on days 1 and 2 post weaning. Data are presented as emmean ± SEM. Non-Dry, non-challenged, standard dry feed, *n* = 8; Ch-Dry, challenged, standard dry feed, *n* = 14; Non-Ferm, non-challenged, fermented liquid feed, *n* = 8; Ch-Ferm, challenged, fermented liquid feed, *n* = 16.

The lowest fecal DM% levels were measured from day 3 to 5 in the Ch-Dry group, from day 2 to 6 in the Ch-Ferm group, whereas DM% in the Non-Ferm group reached its minimum on day 8 (Figure 3). Yet there was no significant difference in DM% between any of the treatment groups during the whole experimental period (*p* = 0.36).

### 3.4. Fecal shedding

When analyzing the probability of ETEC F4 being present in feces based on plate counting, a significant effect of treatment (*p* < 0.001), as well as day (*p* < 0.001), with a significant interaction between group and day (*p* = 0.0149) was observed (Supplementary Figure S1). Validating the challenge model, odds ratio showed that after the first challenge, i.e., on day 2 post weaning, the Ch-Ferm group had 154 times higher risk of having ETEC F4 present in feces compared with the Non-Ferm group (*p* = 0.0277); on day 3 post weaning, the Ch-Dry group had 79 times higher risk of having ETEC F4 present in feces compared with the Non-Dry group (*p* = 0.0011). Both challenged groups had significantly higher risk of having ETEC F4 present in feces compared with non-challenged pigs from day 3 to day 5 post weaning (*p* < 0.05). On day 6 post weaning, while Ch-Dry group had higher risk of having ETEC F4 in feces compared to Non-Dry (*p* = 0.0212) and Non-Ferm (*p* = 0.0156) groups, the Ch-Ferm group had no significant difference with any of the two non-challenged groups (*p* > 0.5). The raw data (Supplementary Figure S2) clearly showed that the two challenged groups had similar level of ETEC shedding while the two non-challenged groups had much lower level.

**Figure 4:**
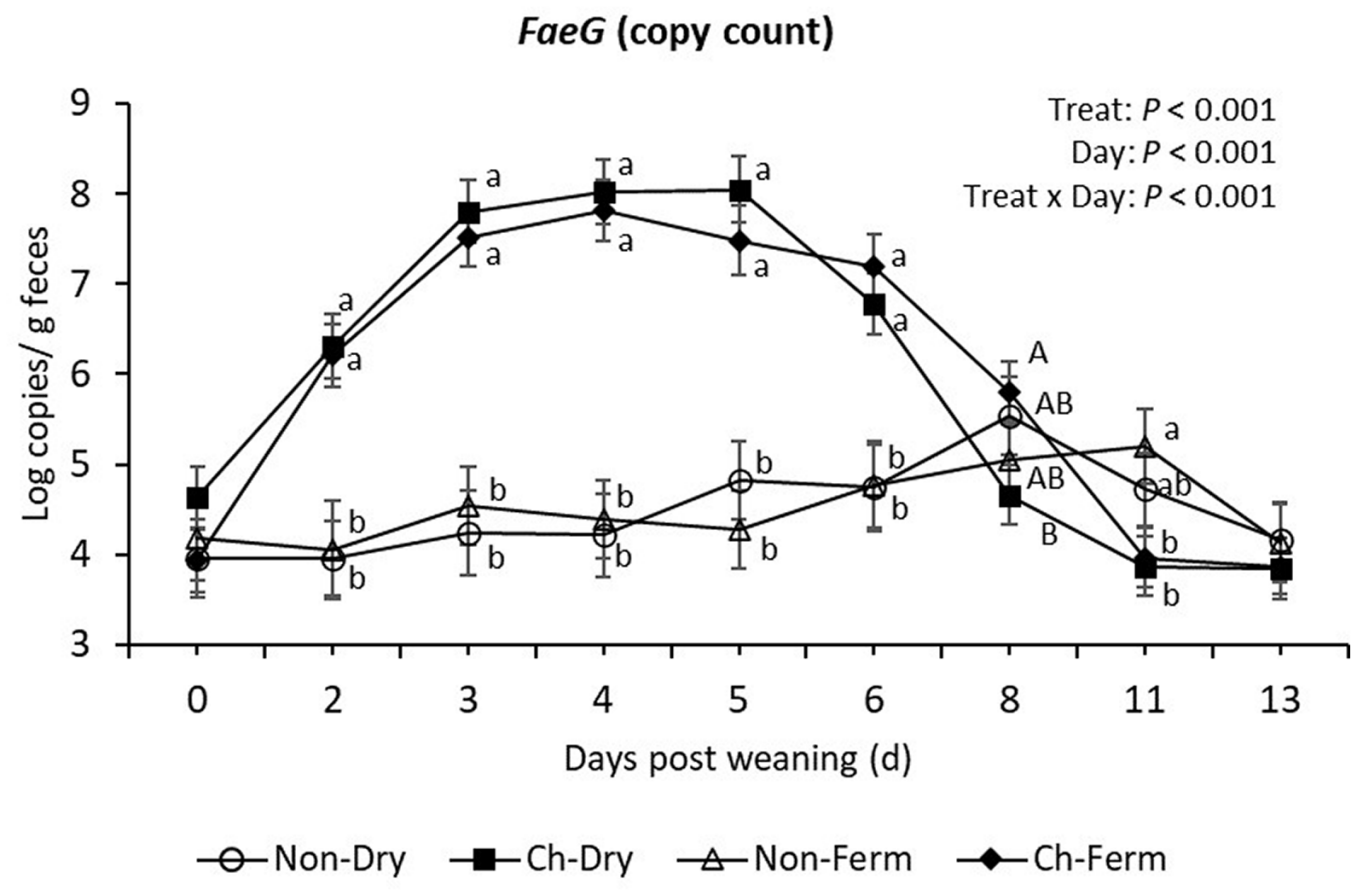
*FaeG* (F4 fimbriae) level in feces (log copies/g) in treatment groups. The limit of detection is 3.8 log copies/g sample. The ETEC F4 was orally administered on days 1 and 2 post weaning. Data are presented as emmean ± SEM. Non-Dry, non-challenged, standard dry feed, *n* = 8; Ch-Dry, challenged, standard dry feed, *n* = 14; Non-Ferm, non-challenged, fermented liquid feed, *n* = 8; Ch-Ferm, challenged, fermented liquid feed, *n* = 16. ^a,b^Indicate statistical significance (*p* < 0.05). ^A,B^Indicate a statistical tendency between treatment groups (0.05 < *p* < 0.10).

From day 2 to day 6, the two challenged groups had significantly higher levels of *FaeG* gene (ETEC F4 fimbriae) compared to the two non-challenged groups (*p* < 0.01), yet there was no significant difference when comparing the Ch-Dry group and Ch-Ferm group (Figure 4). The Ch-Ferm group tended to have higher *FaeG* gene copy numbers compared with the Ch-Dry group on day 8 (*p* = 0.064). On day 11, the Non-Ferm group showed higher copy numbers than the Ch-Dry (*p* = 0.045) and the Ch-Ferm (*p* = 0.034).

**Figure 5:**
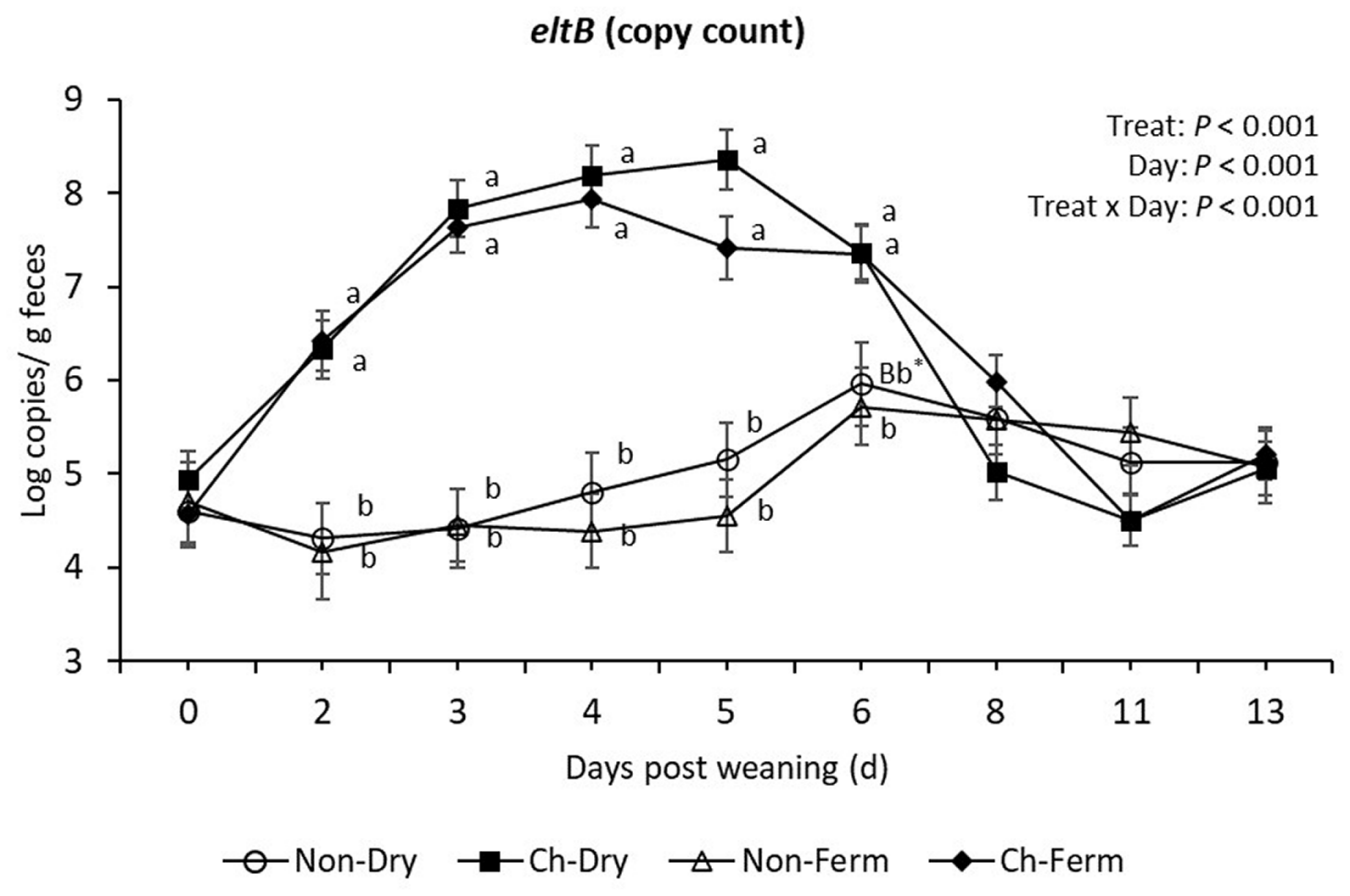
*EltB* (LT toxin) level in feces (log copies/g) in treatment groups. The limit of detection is 3.8 log copies/g sample. The ETEC F4 was orally administered on days 1 and 2 post weaning. Data are presented as emmean ± SEM. Non-Dry, non-challenged, standard dry feed, *n* = 8; Ch-Dry, challenged, standard dry feed, *n* = 14; Non-Ferm, non-challenged, fermented liquid feed, *n* = 8; Ch-Ferm, challenged, fermented liquid feed, *n* = 16. ^a,b^Indicate statistical significance (*p* < 0.05). ^*Bb^Indicates there is a statistical tendency when comparing Non-Dry to Ch-Dry and Chy-Ferm, but there is no difference between Non-Dry and Non-Ferm on day 6.

The number of *eltB* gene (LT toxin) copies from day 2 to day 5 was similar to that of the *FaeG* gene (Figure 5). On day 6, the Non-Ferm group had significantly lower *eltB* gene copy numbers compared to Ch-Ferm (*p* = 0.011) and Ch-Dry (*p* = 0.009), whereas the Non-Dry group tended to be lower than Ch-Dry (*p* = 0.056) and Ch-Ferm (*p* = 0.066).

**Figure 6:**
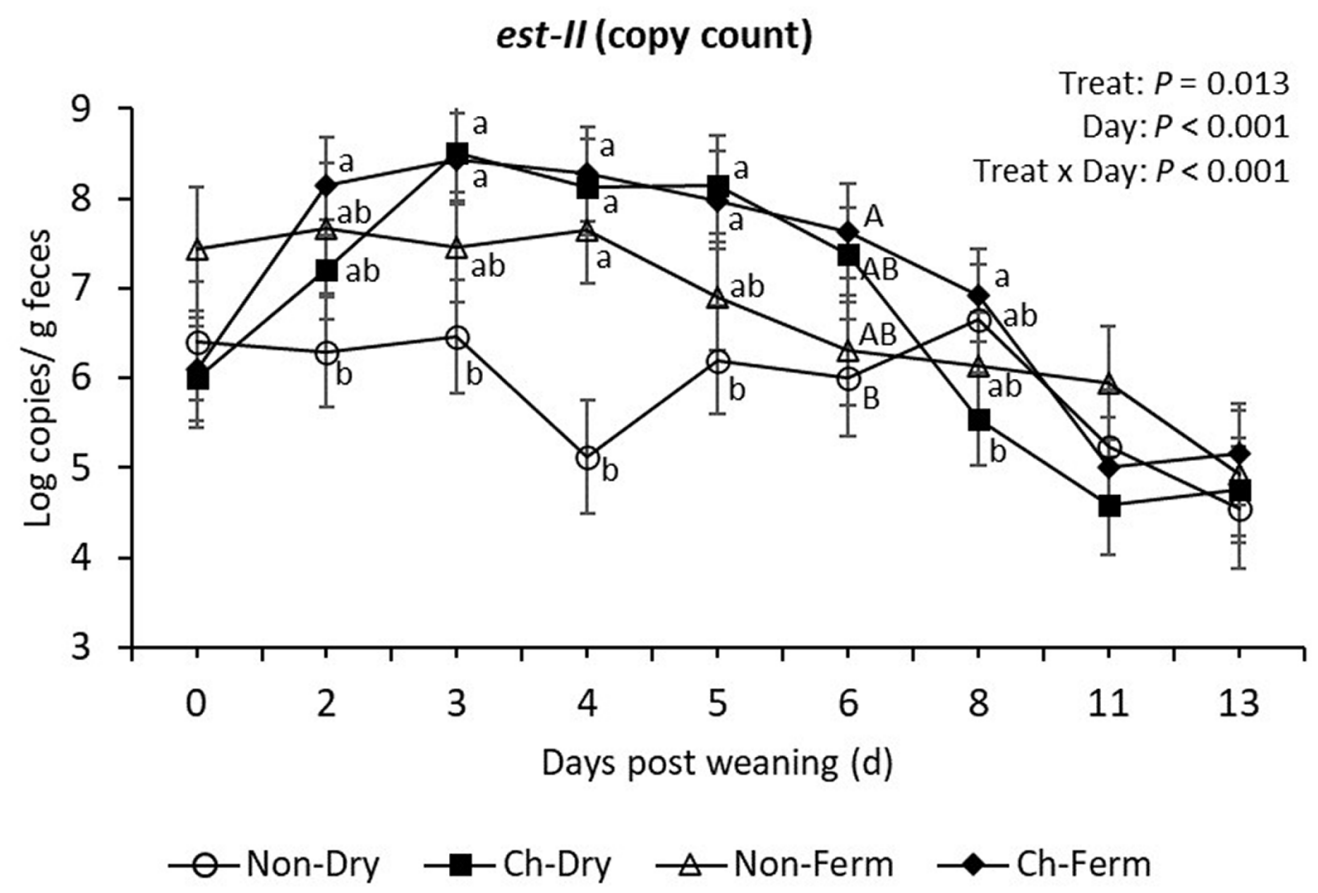
*Est-II* (STb toxin) level in feces (log copies/g) in treatment groups. The limit of detection is 3.8 log copies/g sample. The ETEC F4 was orally administered on days 1 and 2 post weaning. Data are presented as emmean ± SEM. Non-Dry, non-challenged, standard dry feed, *n* = 8; Ch-Dry, challenged, standard dry feed, *n* = 14; Non-Ferm, non-challenged, fermented liquid feed, *n* = 8; Ch-Ferm, challenged, fermented liquid feed, *n* = 16. ^a,b^Indicate statistical significance (*p* < 0.05). ^A,B^Indicate a statistical tendency between treatment groups (0.05 < *p* < 0.10).

The *est-II* gene (heat-stabile toxin STb) was detectable in all treatment groups before challenge, with no significant difference among groups on day 0 (Figure 6). The two challenged groups responded to the ETEC challenge as shown by the increased shedding of *est-II* gene on day 2 and 3 post weaning. The two challenged groups had significantly higher level of *est-II* gene compared to Non-Dry group from day 2 to day 5 post weaning (*p* < 0.05), while the values were not different to those in the Non-Ferm group (*p* > 0.10).

**Figure 7:**
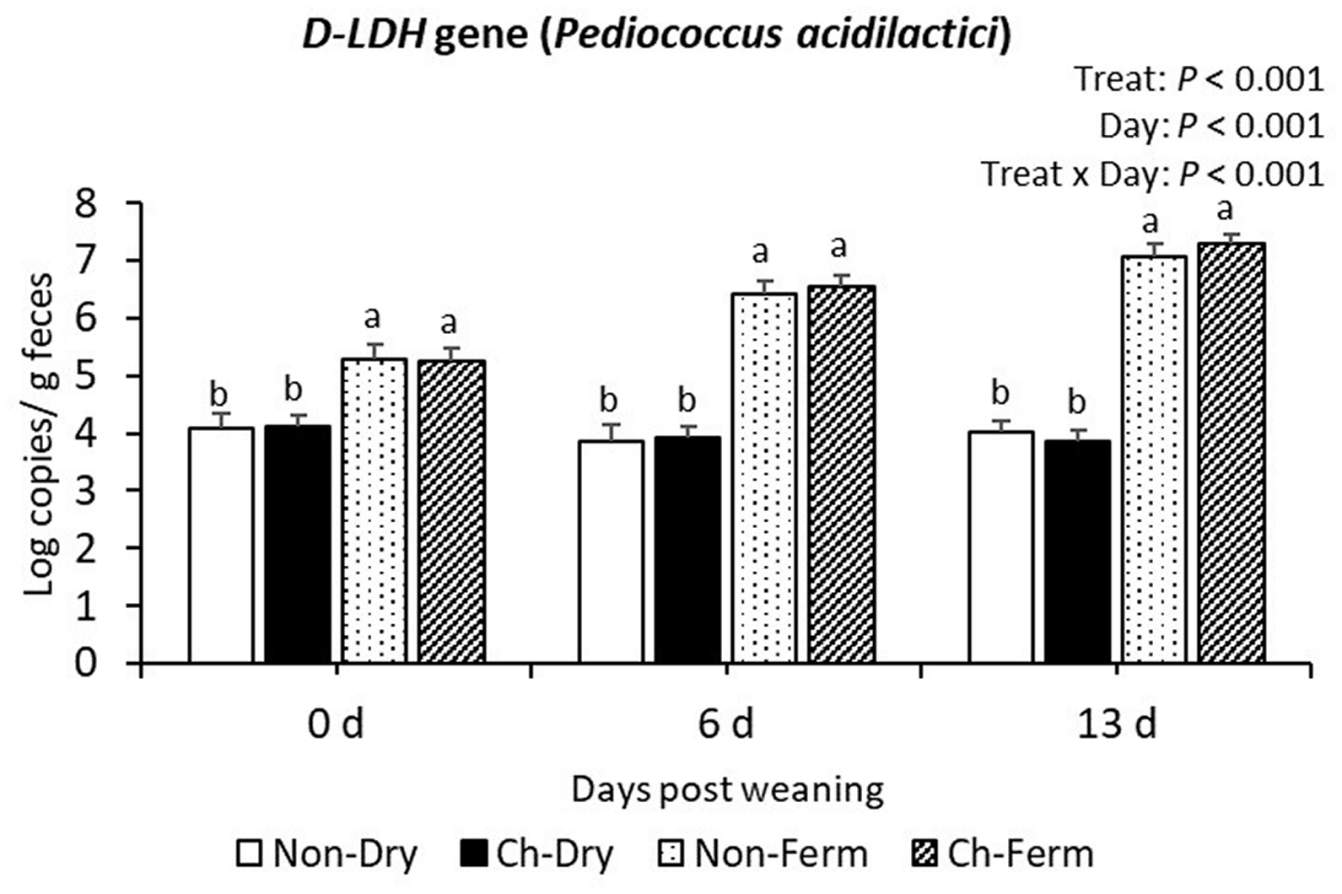
*D-LDH* gene level in feces (log copies/g) in treatment groups. The limit of detection is 3.8 log copies/g sample. The ETEC F4 was orally administered on days 1 and 2 post weaning. Data are presented as emmean ± SEM. Non-Dry, non-challenged, standard dry feed, *n* = 8; Ch-Dry, challenged, standard dry feed, *n* = 14; Non-Ferm, non-challenged, fermented liquid feed, *n* = 8; Ch-Ferm, challenged, fermented liquid feed, *n* = 16. ^a,b^Indicate statistical significance (*p* < 0.05).

On day 0, 6, and 13 post weaning, the two groups fed with FLF had higher copy numbers of *D-LDH* gene (*P. acidilactici*) than the two groups fed with dry feed (*p* < 0.001; Figure 7).

### 3.5. Hematology

Overall, no significant differences between treatment groups were found for hematology parameters, the treatment did not have significant effect on these parameters (*p* > 0.10; Supplementary Table S2). A significant effect of day (*p* < 0.05) was observed in all parameters except red blood cells and eosinophils. Reticulocytes decreased from day 0 (*p* < 0.01) and kept the level from day 4 to day 14. White blood cells and lymphocytes increased from day 0 to day 4 (*p* < 0.01), and from day 4 to day 14 (*p* < 0.01). Significant interaction between treatment and day was detected for red blood cells (*p* = 0.04) and eosinophils (*p* < 0.01).

### 3.6. Immune response parameters

#### 3.6.1. Acute phase proteins

No significant differences in the concentration of plasma CRP between groups were found (Table 5). All groups had low CRP concentrations on day 0, with values ranging from 4.6 to 15.7 μg/mL. A significant effect of day (*p* < 0.001) was observed, with CRP concentration increasing on day 4 in all groups. Overall, the Non-Dry group had significantly lower haptoglobin concentration in plasma than the Ch-Ferm group (*p* = 0.034). The concentration of haptoglobin was affected by day (*p* < 0.001), with values increasing on day 4, and then being reduced on day 14 in all groups, though at higher levels than on day 0.

**Table 5:** Effect of ETEC F4 challenge and fermented liquid feed on acute phase proteins (CRP, Haptoglobin) concentration in plasma^1^.

Item	Treatment^2^				SEM^3^	*	*p*-value		
	Non-Dry	Ch-Dry	Non-Ferm	Ch-Ferm					
							Treat	Day	Treat × Day
C-reactive protein (μg/mL)							0.34	<0.001	0.14
Day 0^4^	4.6	9.5	15.7	7.1	4.1	b			
Day 4	23.4	36.5	40.3	9.4	12.4	a			
Day 14	26.1	30.1	33.3	51.3	15.2	a			
Haptoglobin (mg/mL)							0.03	<0.001	0.11
Day 0	0.2	0.6	0.8	0.9	0.2	c			
Day 4	0.8	1.7	1.7	1.6	0.2	a			
Day 14	0.7	0.9	1.1	1.0	0.1	b			
#	a	ab	ab	b					

1 Values are presented as emmeans.

2 Non-Dry, non-challenged, standard dry feed, *n* = 8; Ch-Dry, challenged, standard dry feed, *n* = 14; Non-Ferm, non-challenged, fermented liquid feed, *n* = 8; Ch-Ferm, challenged, fermented liquid feed, *n* = 16. The ETEC F4 was orally administered on days 1 and 2 post weaning.

3 Pooled standard error of least square means.

4 Day 0: the day of weaning.

*For each parameter, across treatments, values within a column without a common superscript differ between days (*p* < 0.05). #For haptoglobin, across days, values within a row without a common superscript differ between treatments (*p* < 0.05).

#### 3.6.2. Cytokines

Overall, no significant differences between groups were observed for plasma cytokine concentrations of IFN-γ, IL-1β, IL-6, IL-10, IL-12, and TNF-α (*p* > 0.05; Table 6). Significant interaction between treatment and day was detected for IL-12 (*p* = 0.048), which tended to be higher in the Ch-Ferm group compared with the Ch-Dry group on day 0 (*p* = 0.08) and on day 4 (*p* = 0.09), but no difference between treatment groups were seen on day 14. The concentration of IL-6 (*p* < 0.01), IL-10 (*p* < 0.05) and TNF-α (*p* < 0.01) was lower on day 14 compared to day 0 and day 4.

**Table 6:** Effect of ETEC F4 challenge and fermented liquid feed on cytokine concentration in plasma^1^.

Item	Treatment^2^				SEM^3^	*	*p*-value		
	Non-Dry	Ch-Dry	Non-Ferm	Ch-Ferm					
							Treat	Day	Treat × Day
IFN-γ (ng/mL)							0.87	<0.001	0.04
Day 0^4^	28.5	22.7	29.5	27.5	2.4				
Day 4	24.1	24.9	22.7	23.5	2.3				
Day 14	17.9	16.0	14.5	15.4	2.1				
IL-1β (pg/mL)							0.82	0.11	0.54
Day 0	206.4	175.2	211.0	175.2	91.4				
Day 4	160.1	188.5	138.8	180.6	75.2				
Day 14	117.7	162.4	90.6	169.9	63.1				
IL-6 (pg/mL)							0.69	<0.001	0.29
Day 0	165.9	132.5	189.3	162.7	71.3	a			
Day 4	121.0	140.8	106.3	163.0	56.4	a			
Day 14	69.1	77.9	57.0	109.0	36.0	b			
IL-10 (pg/mL)							0.73	0.008	0.41
Day 0	612	492	795	678	305	a			
Day 4	469	509	457	706	242	a			
Day 14	335	361	277	445	173	b			
IL-12 (pg/mL)							0.17	0.69	0.048
Day 0	1,126^AB^	898^B^	1,121^AB^	1,153^A^	87				
Day 4	942^AB^	938^B^	1,093^AB^	1,185^A^	86				
Day 14	926	1,198	1,039	1,154	101				
TNF-α (pg/mL)							0.92	<0.001	0.09
Day 0	114.0	110.8	119.1	110.6	35.9	a			
Day 4	87.7	113.1	82.1	93.2	28.6	a			
Day 14	86.2	52.6	66.2	42.5	18.8	b			

*For each parameter, across treatments, values within a column without a common superscript differ between days (*p* < 0.05).

1 Values are presented as emmeans.

2 Non-Dry, non-challenged, standard dry feed, *n* = 8; Ch-Dry, challenged, standard dry feed, *n* = 14; Non-Ferm, non-challenged, fermented liquid feed, *n* = 8; Ch-Ferm, challenged, fermented liquid feed, *n* = 16. The ETEC F4 was orally administered on days 1 and 2 post weaning.

3 Pooled standard error of least square means.

4 Day 0: the day of weaning.

AB Values within a row without a common superscript tend to differ (0.05 < *p* < 0.1).

### 3.7. Diamine oxidase activity and lipopolysaccharide concentration in plasma

**Figure 8:**
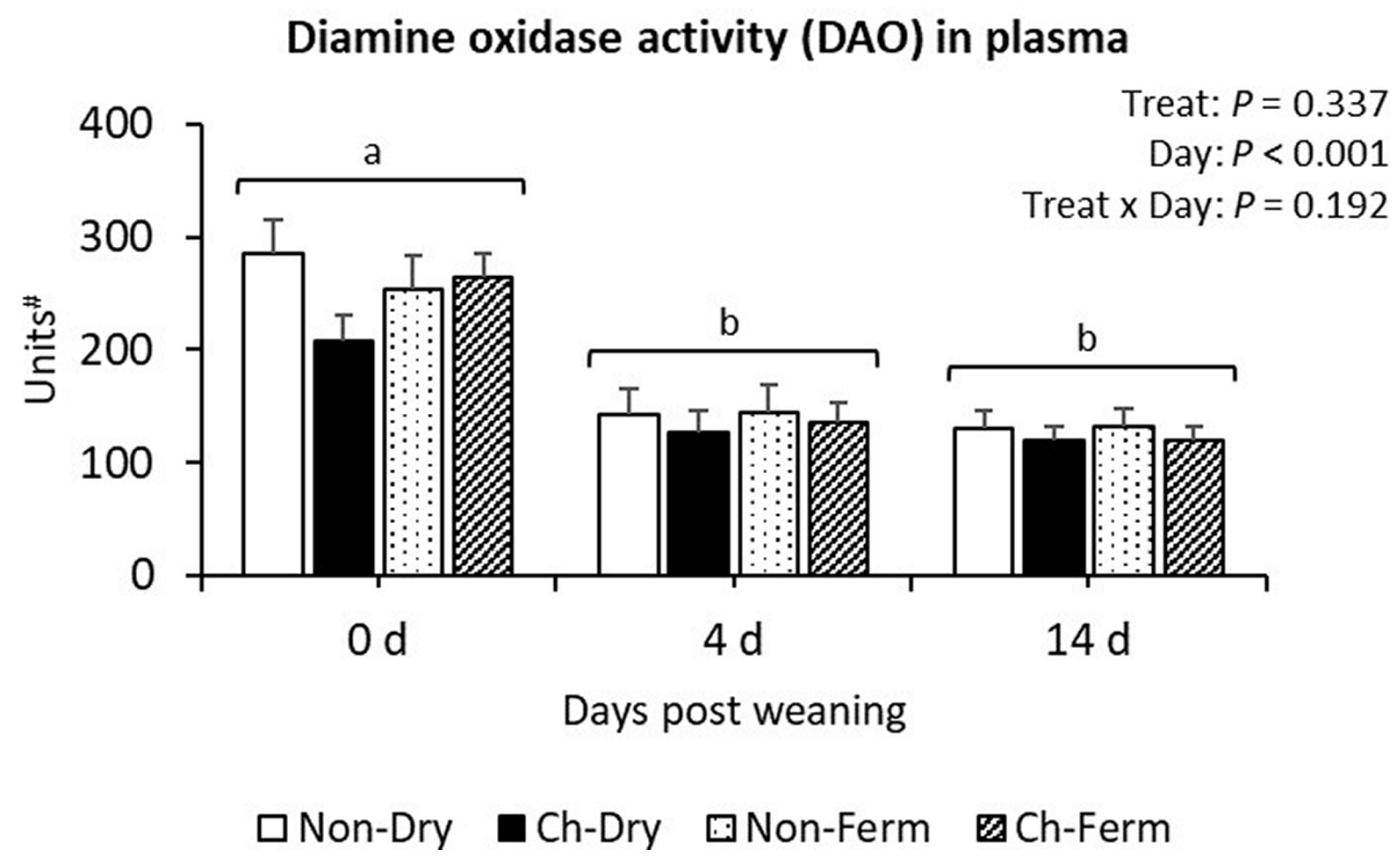
Diamine oxidase activity in plasma (units) in treatment groups. The ETEC F4 was orally administered on days 1 and 2 post weaning. Data are presented as emmean ± SEM. Non-Dry, non-challenged, standard dry feed, *n* = 8; Ch-Dry, challenged, standard dry feed, *n* = 14; Non-Ferm, non-challenged, fermented liquid feed, *n* = 8; Ch-Ferm, challenged, fermented liquid feed, *n* = 16. #: Units were defined as d-emission per min at 590 nm after excitation at 544 nm/80 μL plasma.

Diamine oxidase activity in plasma was similar in all treatment groups (*p* = 0.34; Figure 8). A significant effect of day (*p* < 0.001) was observed, with the concentrations being reduced from day 0 to day 4 and remaining stable on day 14 in all groups.

**Figure 9:**
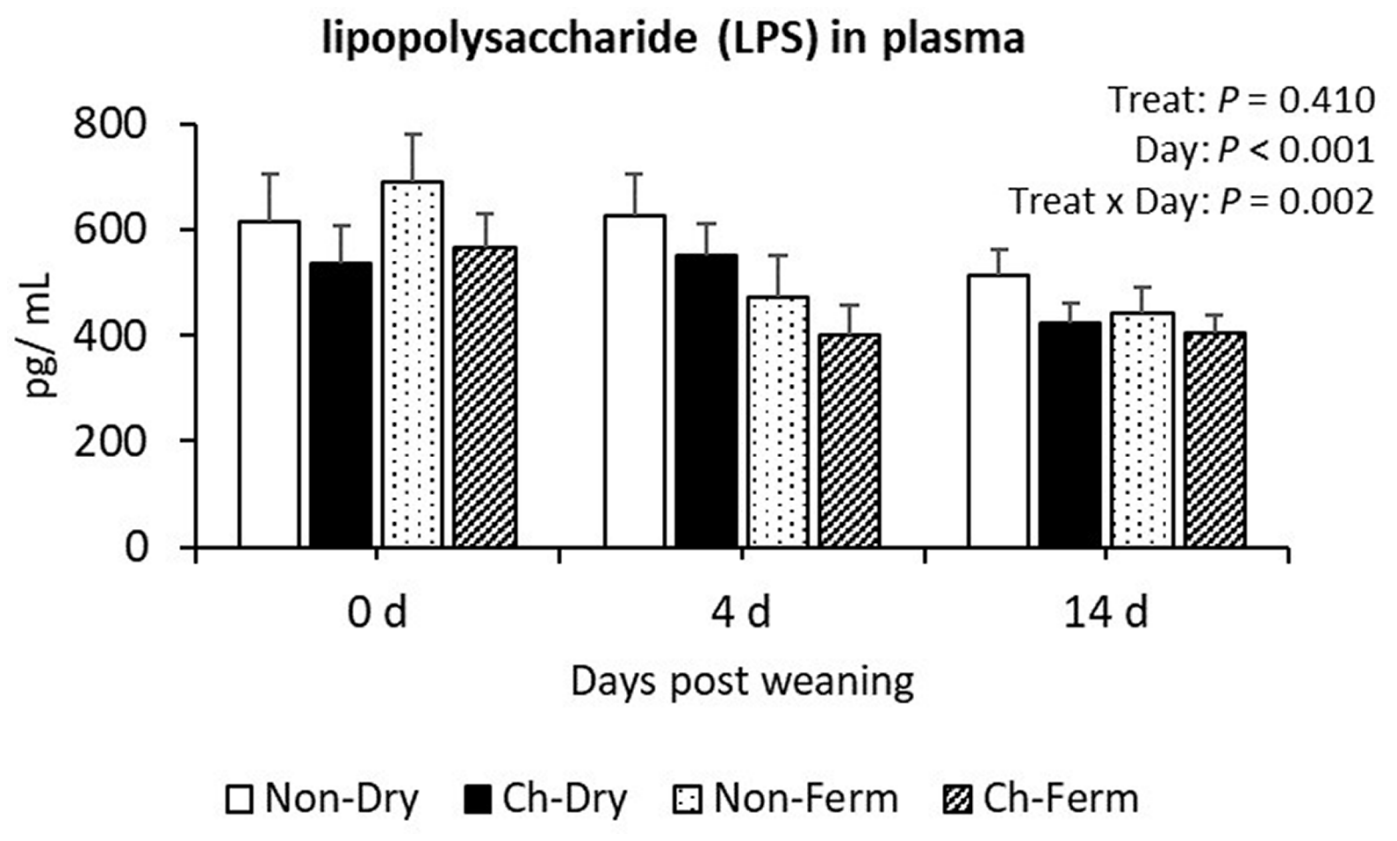
Lipopolysaccharide (LPS) concentration in plasma (pg/mL) in treatment groups. The ETEC F4 was orally administered on days 1 and 2 post weaning. Data are presented as emmean ± SEM. Non-Dry, non-challenged, standard dry feed, *n* = 8; Ch-Dry, challenged, standard dry feed, *n* = 14; Non-Ferm, non-challenged, fermented liquid feed, *n* = 8; Ch-Ferm, challenged, fermented liquid feed, *n* = 16.

No significant differences between treatment groups were found in the plasma lipopolysaccharide (LPS) concentration (Figure 9). There was an interaction between treatment and day (*p* = 0.002), which could be explained by a reduced concentration in the Non-Ferm group with time. On day 0, the Non-Ferm group was numerically higher than other three groups, but on day 4, this group had numerically lower values than the Non-Dry and Ch-Dry groups. All treatment groups had numerically lower values with time after weaning, though.

## 4. Discussion

### 4.1. Feed

According to Brooks et al. (40), pH above 4.5 in FLF can lead to undesirable fermentation as a result of proliferation of certain enteropathogens, i.e., coliforms and *Salmonella* sp. The observed pH value of the fermented cereals in this study was below 4, which is in line with values obtained by Canibe et al. (12) when fermenting liquid cereal grains. Previous studies in our lab have shown great impact of temperature on the fermentation dynamics. Further, an incubation temperature of 30°C was proven preferable by Brooks et al. (41) since it allows a quick production of the desired lactic acid and a fast exclusion of enteropathogens. The large impact of temperature was also shown by Carlson et al. (42) and Beal et al. (43) when fermenting the whole feed. The increase in pH after mixing the remaining feed components with the fermented cereals (~0.9 pH units) was expected and due to the feed components added to the mixing tank, such as calcium carbonate and soybean meal having higher buffering capacity than grain (44). In the present study, the prepared FLF was offered to piglets during the following 24 h, which would allow further fermentation. To reduce the further fermentation and stabilize the liquid feed pH, we added 0.5% benzoic acid to the feed in the present study. Also, adding 0.5% benzoic acid is a rather common strategy in weaner diets as Canibe et al. (45) reviewed. The pH value of the FLF dropped slightly from around 4.7 after mixing the complete feed to 4.4 after 24 h in the mixing tanks, which indicated some fermentation occurred during this period. Microbial metabolite concentrations also indicated that some fermentation had taken place.

As expected, fermentation of the cereals led to lower levels of Enterobacteriaceae, and more than 600 times higher numbers of lactic acid bacteria. Similar findings were reported by Geary et al. (18) and Niven et al. (13) when fermenting the whole feed. The lactic acid concentration in the fermented cereals and FLF in the present study was lower than FLF in other studies where the whole feed was fermented (7, 9, 43), this was also observed by Canibe et al., who attributed this to the low pH value of fermented cereals is likely to inhibit the growth of lactic acid bacteria, thus reducing acid production (12).

Biogenic amines are derived from microbial decarboxylation of amino acids (46); and when present in high concentrations in the feed, they are alleged to contribute to impaired palatability (14). In the present study, biogenic amines were formed but at relatively low level as compared to FLF produced by fermenting the whole feed (12, 13, 47), which was expected, as only cereals were fermented.

In accordance with these results, the levels of amino acids were very similar in the dry feed and FLF (Table 2), indicating no amino acid degradation during fermentation of the cereals and during storage of the FLF. This is in line with the results of Canibe et al. (12) and Pedersen et al. (48). Canibe et al. (12) considered that the fermentation of only grains minimized the degradation of free amino acids. The addition of benzoic acid most probably also contributed to minimizing the fermentation of the complete feed and preventing microbial degradation of free amino acid (49, 50). Another contributing factor might come from *P. acidilactici*, which was reported to result in considerable reduction in lysine metabolism by *E. coli* (13).

Acetic acid is also considered to contribute to impaired palatability at high concentrations, although according to Canibe et al. (51) very high levels would be needed to significantly reduce feed intake. Also, high numbers of yeasts were reported as possible contributors to impaired palatability due to production of “off-flavours” and ethanol (52). The levels of acetic acid (<25 mmol/kg), yeasts (~ 5.5 log CFU/g), and biogenic amines (<130 mg/kg in total) as mentioned above, were not high in FLF in the present study compared to the studies mentioned above, this might explain feed intake was not adversely affected.

Hence, the microbial composition, concentration of microbial metabolites and nutrient composition indicated that the quality of the FLF was high during the whole period the pigs received it.

### 4.2. Growth performance

Feeding FLF numerically prevented weight loss in the first week post weaning. On the other hand, a lower feed intake of the Ch-Ferm group compared to the non-challenged groups during the first week post weaning was observed, which could be speculated to be partly due to the DM content of the FLF. The final DM content of FLF had to be reduced (22% in practice vs. 27% as originally planned) due to blockage of the feeding tubes. However, Geary et al. (53) reported no significant effect of dietary DM on DM feed intake, weight gain or feed conversion ratio, because the piglets maintained DM feed intake by increasing their total volumetric feed intake when offered lower DM diet (14.9% DM versus 25.5% DM); meanwhile the piglets fed the lower DM diet reduced their water intake from nipple waterers as compared to the higher DM diet group. In the present study, the water intake from nipple waterers was not recorded. Canibe et al. (12) reported a lower feed intake and average daily gain in piglets fed with feed produced by fermenting the cereals and adding the remaining ingredients immediately before feeding as compared to the same diet fed as dry feed. However, as the authors argued, the two dietary groups could not be compared since the pigs received the dry feed were allowed *ad libitum* access to the feed, whereas the pigs received the feed containing fermented cereals was fed restrictively.

Brooks pointed out that if an initially nutritionally balanced FLF did not affect feed intake, it was likely that the biochemical changes produced by fermentation lead to a less-balanced diet (54). Yet considering the good quality of FLF in the present study as discussed above, this does not seem to explain a lack of expected positive effect of FLF on feed intake. Improvement in feeder design (trough with a sloped bottom and a step up) (55), and the addition of sepiolite to control the sedimentation of the solids in FLF (56), or other factors might be ways of improving the results.

### 4.3. Diarrhea, ETEC F4 and enterotoxin shedding

Generally, ETEC-caused diarrhea in weaned piglets has been difficult to model experimentally because of its multifactorial nature; the combination of factors required to induce diarrhea has not been fully understood (57). For example, Xia et al. (58) reported that, due to transportation and grouping, mild and temporary diarrhea was observed before the bacteria challenge had been conducted. In the present study, we challenged the piglets on day 1 and 2 after weaning, expecting to take advantage of the stressful situation, yet the challenge did not influence the diarrhea prevalence significantly. The lack of response on diarrhea in the present study could be partially attributed to the timing of the challenge, which was very close to the weaning date. This might allow the passive immunity derived from sow’s milk to assist piglets in facing the infection (21). However, both the detected probability of ETEC F4 strain in feces, and the elevated fecal shedding of F4 fimbriae and enterotoxin observed in the challenged pigs but not in the non-challenged pigs indicated the validity of the ETEC challenge model (21). This demonstrated that although piglets shed ETEC at high levels, there may not necessarily be obvious diarrhea and vice versa. This agrees well with other studies (59–61) and indicates that several response parameters should be included in this type of challenge models.

As reviewed by Lauridsen et al. (62), very few published studies have been designed to test the impact of FLF on PWD, and therefore it is difficult to conclude on it. Missotten et al. (56) and Pedersen (63) reported no effect of FLF, produced by fermenting the whole feed, on diarrhea compared with control dry feed in weaned pigs, Pedersen (48) and Maribo et al. (64) reported no effect of FLF, produced by fermenting the cereal fraction, on the frequency of diarrhea treatment compared with non-FLF in pigs at different ages. There are few studies evaluating the effect of FLF on PWD under controlled ETEC infection settings as the present study, and with limited number of repetitions. In two challenge studies with *E. coli* O149: F4, feeding diets including whey (61) or whey permeate (65) fermented with lactic acid bacteria, did not affect the severity or frequency of PWD. In the present study, FLF had no influence on the probability of developing diarrhea compared to dry feed, either under challenge or non-challenged conditions. It should be kept in mind that, as shown above, the challenged pigs developed diarrhea to a very low extent, which makes it difficult to conclude on this parameter.

In general, according to the characteristics of FLF as measured here, a lower ETEC fecal shedding and/or faster clearance was expected when feeding piglets with FLF. Overall, quantitative detection of genes showed that FLF did not influence the level of ETEC F4 or enterotoxin in feces in the present study. The findings support the experiment where Missotten et al. (56) reported that FLF did not affect fecal coliform counts. However, the effect of FLF on the Enterobacteriaceae is not always consistent. Some studies reported that FLF reduced fecal shedding of Enterobacteriaceae (66), and the number of Enterobacteriaceae (9), *E. coli* (67) and coliforms (6) in the rectum. It is worth noting that the pigs in these studies were not challenged with ETEC. Unlike the findings in our study, Amezcua et al. (61) reported that pigs fed FLF eliminated the bacteria slower than the control dry feed group under ETEC challenge. It is difficult to speculate on the reasons for the absence of expected results, but high doses of probiotics may not always yield positive results, as previously reported (68).

We found a good correlation between the two methods of measuring ETEC F4 in feces (microbiological enumeration and qPCR), yet qPCR showed a higher number of detectable *FaeG* gene encoding for the F4 fimbriae than the detectable bacterial from the microbiological enumeration. This finding is in line with the study conducted by Hansen et al. (33), with ETEC F18 being measured in that study. The qPCR method detects all cells in a sample, including the dead cells or the DNA of bacteria in the environment (69), whereas when plate counting, only viable cells that are capable of forming colonies on nutrient media are counted. Nonetheless, the present study shows that quantifying virulence genes is a useful method for assessing ETEC shedding in weaned pigs.

The *est-II* gene encoding for STb toxin was detectable at relatively high levels in all treatment groups before ETEC challenge in the present study. This is consistent with the findings in previous research (33, 70–72). The presence of the *est-II* gene is most probably associated with non-hemolytic *E. coli*, since quantification of the *FaeG* gene and bacterial plate counting followed by serotyping confirmed that neither ETEC O149:F4 nor other hemolytic *E. coli* were detected (except three pigs in the Ch-Dry in the first block, which showed low levels of *FaeG* gene) before ETEC challenge. The *eltB* gene encoding for LT toxin was detectable before ETEC challenge in all treatment groups in the second block of the present study, though at relative low levels. With undetectable level of *FaeG* gene in the second block as mentioned above, this finding may indicate that pigs used in the second block had some level of LT-positive, F4-negative *E. coli*. Contrary to our findings, Rhouma et al. (71) did not find the gene encoding LT in feces before challenge; however, the detection limit was not indicated. It’s interesting to notice that both the study of Rhouma et al. (71) and our study showed similar profiles of *eltB* and *FaeG* gene levels after challenge, and also more animals shed detectable levels of *eltB* gene than of *FaeG* gene. Since the piglets did not suffer from diarrhea before the ETEC challenge, these findings indicate that the presence of enterotoxin-positive but F4 fimbriae-negative *E. coli* at these levels do not cause diarrhea.

### 4.4. Blood parameters

Overall, the plasma concentration of acute phase proteins (CRP and haptoglobin) increased after weaning in the present study. This result is in line with earlier literature (73) pointing out that induction of stress through mixing animals or changes in feeding patterns may cause this increase. We observed a great individual variability, which was previously reported by Pomorska-Mól (73) and Yu et al. (74), indicating the difference in reactions to stress among individuals. Our experience with acute phase proteins analyses indicate variation from batch to batch of pigs used in ETEC challenge studies conducted in the same facility (33). Moreover, the acute phase proteins reference ranges/threshold values for ETEC infection are still unclear due to different methods applied in different institutions. Therefore, it could be difficult to compare acute phase proteins actual values between different studies.

Several studies have demonstrated that weaning itself can initiate intestinal inflammation (75) and is closely related to oxidative stress (76, 77). During inflammation, locally produced cytokines may reach detectable levels at systemic level (78). Our data showed that the plasma TNF-α, IL-6 and IL-10 levels decreased from day 4 to day 14 post weaning in all groups, and a similar profile, although not significant, was observed for IFN-γ. These results may indicate that inflammatory reactions, which were induced by weaning, were ameliorated with time. Since studies in humans have shown increased hematocrit levels after stress (79), the decreased hematocrit levels from day 4 to day 14 observed here also provided indications of this weaning stress recovery, yet this value, together with other hematology indexes, was within reference intervals for piglets of this age (80, 81). González-Ramón et al. (82) reported that pig haptoglobin was mainly regulated by IL-6, which is in good agreement with the results of the present study, as we observed a decrease in both IL-6 and haptoglobin from day 4 to day 14 post weaning.

Kim et al. (83) reviewed that the peak of systemic inflammation with regard to pro-inflammatory cytokines and acute phase proteins occurred on day 2 to 7 post ETEC challenge in weaned pigs, and after day 14 post challenge it became undetectable. The ETEC challenge in the present study did not elevate pro-inflammatory cytokines significantly, though. Moreover, the plasma TNF-α level of the challenged groups was lower in the present study compared with other ETEC challenge studies conducted on post-weaning pigs (84, 85). This finding, suggesting a low level of systemic inflammation, could partially be attributed to the time of sampling (3 h/6 h/12 h post challenge (84) vs. 3 days post challenge in our study). It is worth noting that the TNF-α level of all groups before challenge (>110 pg./mL) and the value in non-challenged groups in the present study (>80 pg./mL on day 4) are higher than in the mentioned studies. This difference might partially be due to the time between weaning and challenge. Lee et al. (85) allowed a 7-day adaptation period before challenge, and Li et al. (84) 15 days, whereas in the present study, the piglets were challenged 1 and 2 days after weaning. López-Colom pointed out that the baseline values of TNF-α was around ≤100 pg./mL in weaned piglets as a biomarker in digestive pathologies (86). Our results may indicate that all the pigs in the present study experienced some degree of systemic inflammation before or at weaning. Confirming this, our data shows a reduction of plasma DAO, a marker for gut epithelial barrier, from the weaning day, indicating that the intestinal epithelial barrier function was gradually improved after weaning. There was no difference in the level of plasma LPS among treatment groups, which suggests no acute disruption of the gut barrier, together with the lack of differences in hematology profile, providing additional evidence for a low level of systemic inflammation caused by ETEC.

## 5. Conclusion

The present study examined the influence of FLF with added *P. acidilactici* on weaned pigs after an experimental ETEC F4:O149 challenge. The results showed no impact of this feeding strategy on diarrhea, ETEC shedding or related parameters. Although the ETEC challenge did not induce clear signs of infection after the challenge, a reduction in the shedding of the inoculated ETEC could be expected, but this was not observed. The study showed that a strategy like this can be a way of providing a high level of probiotics to pigs by allowing their proliferation during fermentation.

## Data availability statement

The original contributions presented in the study are included in the article/Supplementary material, further inquiries can be directed to the corresponding author.

## Ethics statement

The animal study was reviewed and approved by the Danish Animal Experiments Inspectorate, Ministry of Food, Agriculture and Fisheries, Danish Veterinary and Food Administration.

## Author contributions

JX: data curation and analysis, original draft preparation, and review and editing. SN: data curation and analysis and review and editing. CL: experimental design, and review and editing. HL: data curation and analysis, and review and editing. NC: experimental design, data curation and analysis, and review and editing. All authors contributed to the article and approved the submitted version.

## Funding

This work was funded by the Ministry of Food, Agriculture and Fisheries of Denmark. JX acknowledges the scholarship from China Scholarship Council.

## Conflict of interest

The authors declare that the research was conducted in the absence of any commercial or financial relationships that could be construed as a potential conflict of interest.

## Publisher’s note

All claims expressed in this article are solely those of the authors and do not necessarily represent those of their affiliated organizations, or those of the publisher, the editors and the reviewers. Any product that may be evaluated in this article, or claim that may be made by its manufacturer, is not guaranteed or endorsed by the publisher.

## Abbreviations

PWD: post-weaning diarrhea
ETEC: Enterotoxigenic *Escherichia coli*
FLF: fermented liquid feed
*P. acidilactici*: *Pediococcus acidilactici*
CFU: colony-forming unit
DM: dry matter
CRP: C-reactive protein
qPCR: quantitative real-time polymerase chain reaction
DAO: diamine oxidase activity
LPS: lipopolysaccharide
NDO: non-digestible oligosaccharides
NSP: non-starch polysaccharides
NCP: non-cellulosic polysaccharides
LOQ: limit of quantification
ADG: average daily gain
ADFI: average daily feed intake
G:F: gain to feed ratio

## References

[1] Amezcua R Friendship RM Dewey CE Gyles C Fairbrother JM. Presentation of postweaning *Escherichia coli* diarrhea in southern Ontario, prevalence of Hemolytic *E. coli* serogroups involved, and their antimicrobial resistance patterns. Can J Vet Res. (2002) 66:73–8. PMID: 11989737 PMC226986

[2] Nagy B Fekete PZ. Enterotoxigenic *Escherichia coli* in veterinary medicine. Int J Med Microbiol. (2005) 295:443–54. doi: 10.1016/j.ijmm.2005.07.003 16238018

[3] Govindarajan DK Viswalingam N Meganathan Y Kandaswamy K. Adherence patterns of *Escherichia coli* in the intestine and its role in pathogenesis. Med Microecol. (2020) 5:100025. doi: 10.1016/j.medmic.2020.100025

[4] Vipin Madhavan TP Sakellaris H. Chapter five - colonization factors of enterotoxigenic *Escherichia coli* In: Sariaslani S Gadd GM, editors. Advances in Applied Microbiology, vol. 90. Cambridge, Massachusetts, United States: Academic Press (2015). 155–97. 10.1016/bs.aambs.2014.09.003 25596032

[5] López-Gálvez G López-Alonso M Pechova A Mayo B Dierick N Gropp J. Alternatives to antibiotics and trace elements (copper and zinc) to improve gut health and Zootechnical parameters in piglets: a review. Anim Feed Sci Technol. (2021) 271:114727. doi: 10.1016/j.anifeedsci.2020.114727

[6] Mikkelsen LL Jensen BB. Performance and microbial activity in the gastrointestinal tract of piglets fed fermented liquid feed at weaning. J Anim Feed Sci. (1998) 7:211–5. doi: 10.22358/jafs/69978/1998

[7] Canibe N Jensen BB. Fermented and nonfermented liquid feed to growing pigs: effect on aspects of gastrointestinal ecology and growth performance. J Anim Sci. (2003) 81:2019–31. doi: 10.2527/2003.8182019x, PMID: 12926784

[8] Canibe N Jensen BB. Fermented liquid feed—microbial and nutritional aspects and impact on enteric diseases in pigs. Anim Feed Sci Technol. (2012) 173:17–40. doi: 10.1016/j.anifeedsci.2011.12.021

[9] van Winsen RL Urlings BAP Lipman LJA Snijders JMA Keuzenkamp D Verheijden JHM. Effect of fermented feed on the microbial population of the gastrointestinal tracts of pigs. Appl Environ Microbiol. (2001) 67:3071–6. doi: 10.1128/AEM.67.7.3071-3076.2001, PMID: 11425724 PMC92983

[10] Boesen HT Jensen TK Schmidt AS Jensen BB Jensen SM Møller K. The influence of diet on *Lawsonia intracellularis* colonization in pigs upon experimental challenge. Vet Microbiol. (2004) 103:35–45. doi: 10.1016/j.vetmic.2004.06.008, PMID: 15381264

[11] Lindecrona RH Jensen TK Jensen BB Leser TD Jiufeng W Møller K. The influence of diet on the development of swine dysentery upno experimental infection. Anim Sci. (2003) 76:81–7. doi: 10.1017/S1357729800053340

[12] Canibe N Højberg O Badsberg JH Jensen BB. Effect of feeding fermented liquid feed and fermented grain on gastrointestinal ecology and growth performance in piglets. J Anim Sci. (2007) 85:2959–71. doi: 10.2527/jas.2006-744, PMID: 17591711

[13] Niven SJ Beal JD Brooks PH. The effect of controlled fermentation on the fate of synthetic lysine in liquid diets for pigs. Anim Feed Sci Technol. (2006) 129:304–15. doi: 10.1016/j.anifeedsci.2005.12.016

[14] Brooks PH Beal JD Niven S. Liquid feeding of pigs: potential for reducing environmental impact and for improving productivity and food safety. Recent Adv Anim Nutr Aust. (2001) 13:49–63.

[15] Fan P Song P Li L Huang C Chen J Yang W. Roles of biogenic amines in intestinal signaling. Curr. Protein Pept. Sci. (2017) 18, 532–40. doi: 10.2174/1389203717666160627073048 27356940

[16] Pieper R Villodre Tudela C Taciak M Bindelle J Pérez JF Zentek J. Health relevance of intestinal protein fermentation in young pigs. Anim Health Res Rev. (2016) 17:137–47. doi: 10.1017/S1466252316000141, PMID: 27572670

[17] Moran CA Scholten RHJ Tricarico JM Brooks PH Verstegen MWA. Fermentation of wheat: effects of backslopping different proportions of pre-fermented wheat on the microbialand chemical composition. Arch Anim Nutr. (2006) 60:158–69. doi: 10.1080/17450390600562700, PMID: 16649578

[18] Geary TM Brooks PH Beal JD Campbell A. Effect on Weaner pig performance and diet microbiology of feeding a liquid diet acidified to Ph 4 with either lactic acid or through fermentation with *Pediococcus acidilactici*. J Sci Food Agric. (1999) 79:633–40. doi: 10.1002/(SICI)1097-0010(19990315)79:4<633::AID-JSFA231>3.0.CO;2-L

[19] Canibe N Joan Noel S Nygaard LH. P155. Using probiotics and enzymes to improve liquid fermented feed for piglets. Anim Sci Proce. (2022) 13:205. doi: 10.1016/j.anscip.2022.03.358

[20] Tybirk P Sloth NM Kjeldsen N Blaabjerg K. Danish Nutrient Requirement Standards (in Danish: Normer for Næringsstoffer). Copenhagen, Denmark: SEGES Danish Pig Research Centre (2021).

[21] Luise D Lauridsen C Bosi P Trevisi P. Methodology and application of *Escherichia coli* F4 and F18 encoding infection models in post-weaning pigs. J Anim Sci Biotechnol. (2019) 10:53. doi: 10.1186/s40104-019-0352-7, PMID: 31210932 PMC6567477

[22] Cunniff P Washington D. Official methods of analysis of Aoac international. J AOAC Int. (1997) 80:127A–8A. doi: 10.1093/jaoac/80.6.127A

[23] Hansen B. Determination of nitrogen as elementary N, an alternative to Kjeldahl. Acta Agric Scand. (1989) 39:113–8. doi: 10.1080/00015128909438504

[24] European Economic Community. Commission directive 98/64/Ec of 3 September 1998 establishing community methods of analysis for the determination of amino-acids, crude oils and fats, and Olaquindox in feeding stuffs and amending directive 71/393/Eec. Off J Eur Communities. (1998) 257:14–28.

[25] Jensen SK. Improved Bligh and dyer extraction procedure. Lipid Technol. (2008) 20:280–1. doi: 10.1002/lite.200800074

[26] Knudsen KEB. Carbohydrate and lignin contents of plant materials used in animal feeding. Anim Feed Sci Technol. (1997) 67:319–38. doi: 10.1016/S0377-8401(97)00009-6

[27] Vangsøe CT Sørensen JF Bach Knudsen KE. Aleurone cells are the primary contributor to Arabinoxylan oligosaccharide production from wheat bran after treatment with Cell Wall-degrading enzymes. Int J Food Sci Technol. (2019) 54:2847–53. doi: 10.1111/ijfs.14201

[28] Poulsen A-SR Jonge N d Nielsen JL Højberg O Lauridsen C Cutting SM. Impact of *Bacillus* Spp. spores and gentamicin on the gastrointestinal microbiota of suckling and newly weaned piglets. PLoS One. (2018) 13:e0207382. doi: 10.1371/journal.pone.0207382 30481191 PMC6258502

[29] Frydendahl K Imberechts H Lehmann S. Automated 5′ nuclease assay for detection of virulence factors in porcine *Escherichia Coli*. Mol Cell Probes. (2001) 15:151–60. doi: 10.1006/mcpr.2001.0354, PMID: 11352596

[30] Wang W Zijlstra RT Gänzle MG. Identification and quantification of virulence factors of enterotoxigenic *Escherichia Coli* by high-resolution melting curve quantitative PCR. BMC Microbiol. (2017) 17:114. doi: 10.1186/s12866-017-1023-5, PMID: 28506262 PMC5433089

[31] Mora D Fortina MG Parini C Manachini PL. Identification of *Pediococcus acidilactici* and *Pediococcus pentosaceus* based on 16s rRNA and *ldhD* gene-targeted multiplex PCR analysis. FEMS Microbiol Lett. (1997) 151:231–6. doi: 10.1111/j.1574-6968.1997.tb12575.x, PMID: 9228758

[32] Thermo Fisher Scientific. DNA Copy Number and Dilution Calculator; 05 January. (2023). Available at: https://www.thermofisher.com/dk/en/home/brands/thermo-scientific/molecular-biology/molecular-biology-learning-center/molecular-biology-resource-library/thermo-scientific-web-tools/dna-copy-number-calculator.html. (Accessed January 05, 2023).

[33] LHB H Lauridsen C Nielsen B Jørgensen L Canibe N. Impact of early inoculation of probiotics to suckling piglets on postweaning Diarrhoea – a challenge study with enterotoxigenic *E. coli* F18. Animal. (2022) 16:100667. doi: 10.1016/j.animal.2022.100667 36368266

[34] R Core Team. R: A Language and Environment for Statistical Computing. Vienna, Austria: R Foundation for Statistical Computing (2022).

[35] Hartig F. Dharma: Residual Diagnostics for Hierarchical (Multi-Level/Mixed) Regression Models. v0.4.6 ed; (2022).

[36] Lüdecke D Ben-Shachar M Patil I Waggoner P Makowski D. Performance: an {R} package for assessment, comparison and testing of statistical models. J Open Source Soft. (2021) 6:3139. doi: 10.21105/joss.03139

[37] Bates D Mächler M Bolker B Walker S. Fitting linear mixed-effects models using Lme4. J Stat Softw. (2015) 67:1–48. doi: 10.18637/jss.v067.i01

[38] Lee W Grimm KJ. Generalized linear mixed-effects modeling programs in R for binary outcomes. Struct Equ Model Multidiscip J. (2018) 25:824–8. doi: 10.1080/10705511.2018.1500141

[39] Chung Y Rabe-Hesketh S Dorie V Gelman A Liu J. A nondegenerate penalized likelihood estimator for variance parameters in multilevel models. Psychometrika. (2013) 78:685–709. doi: 10.1007/s11336-013-9328-2, PMID: 24092484

[40] Brooks PH Moran C Beal J Demeckova V Campbell A. Liquid feeding for the young piglet In: Varley MA Wiseman J, editors. Weaner Pig: Nutrition and Management. Wallingford, UK: CABI (2001). 153.

[41] Brooks PH. Liquid feeding as a means to promote pig health In: Murphy JM Lange CFM, editors. 3rd London Swine Conference - Maintaining Your Competitive Edge; 9–10 April. Ontario, Canada (2003). 83–103.

[42] Carlson D Poulsen HD. Phytate degradation in soaked and fermented liquid feed—effect of diet, time of soaking, heat treatment, Phytase activity, Ph and temperature. Anim Feed Sci Technol. (2003) 103:141–54. doi: 10.1016/S0377-8401(02)00288-2

[43] Beal JD Niven SJ Campbell A Brooks PH. The effect of temperature on the growth and persistence of Salmonella in fermented liquid pig feed. Int J Food Microbiol. (2002) 79:99–104. doi: 10.1016/S0168-1605(02)00183-6, PMID: 12382689

[44] Lawlor PG Lynch PB Caffrey PJ O’Reilly JJ O’Connell MK. Measurements of the acid-binding capacity of ingredients used in pig diets. Ir Vet J. (2005) 58:447–52. doi: 10.1186/2046-0481-58-8-447, PMID: 21851673 PMC3113917

[45] Canibe N Højberg O Kongsted H Vodolazska D Lauridsen C Nielsen TS. Review on preventive measures to reduce post-weaning Diarrhoea in piglets. Animals. (2022) 12:2585. doi: 10.3390/ani12192585, PMID: 36230326 PMC9558551

[46] Özogul Y Özogul F. Chapter 1 biogenic amines formation, toxicity, regulations in food In: Saad B Tofalo R, editors. Biogenic Amines in Food: Analysis, Occurrence and Toxicity. London, United Kingdom: The Royal Society of Chemistry (2020). 1–17.

[47] Jakobsen GV Jensen BB Bach Knudsen KE Canibe N. Fermentation and addition of enzymes to a diet based on high-moisture corn, rapeseed cake, and peas improve digestibility of nonstarch polysaccharides, crude protein, and phosphorus in pigs. J Anim Sci. (2015) 93:2234–45. doi: 10.2527/jas.2014-8644, PMID: 26020320

[48] Pedersen A. Fermenteret Korn Til Smågrise. Report 728. Copenhagen, Denmark: Landsudvalget for Svin (2006).

[49] Vils E Canibe N Sommer HM. Benzoic Acid Inhibits the Degradation of Free Amino Acids in Liquid Feed. Trial Report 1156. Copenhagen, Denmark: SEGES Danish Pig Research Centre (2018).

[50] O’Meara FM Gardiner GE O’Doherty JV Lawlor PG. Effect of dietary inclusion of benzoic acid (Vevovitall®) on the microbial quality of liquid feed and the growth and carcass quality of grow-finisher pigs. Livest Sci. (2020) 237:104043. doi: 10.1016/j.livsci.2020.104043

[51] Canibe N Pedersen AØ Jensen BB. Impact of acetic acid concentration of fermented liquid feed on growth performance of piglets. Livest Sci. (2010) 133:117–9. doi: 10.1016/j.livsci.2010.06.040

[52] Jensen B Mikkelsen L. Feeding liquid diets to pigs In: Garnsworthy PC Wiseman J, editors. Recent Advances in Animal Nutrition. Nottingham: Nottingham University Press (1998). 107–26.

[53] Geary TM Brooks PH Morgan DT Campbell A Russell PJ. Performance of weaner pigs fedad libitum with liquid feed at different dry matter concentrations. J Sci Food Agric. (1996) 72:17–24. doi: 10.1002/(SICI)1097-0010(199609)72:1<17::AID-JSFA598>3.0.CO;2-3

[54] Brooks PH. Fermented liquid feed for pigs. CAB reviews: perspectives in agriculture, veterinary science. Nutr Nat Resourc. (2008) 2008:1–18. doi: 10.1079/PAVSNNR20083073

[55] Lawlor PG Lynch PB Gardiner GE Caffrey PJ O’Doherty JV. Effect of liquid feeding weaned pigs on growth performance to harvest 1,2. J Anim Sci. (2002) 80:1725–35. doi: 10.2527/2002.8071725x 12162639

[56] Missotten J Michiels J Ovyn A De Smet S Dierick N. Fermented liquid feed for weaned piglets: impact of sedimentation in the feed slurry on performance and gut parameters. Czech J Anim Sci. (2015) 60:195–207. doi: 10.17221/8169-CJAS

[57] Jensen G Frydendahl K Svendsen O Jorgensen C Cirera S Fredholm M. Experimental infection with *Escherichia coli* O149:F4ac in weaned piglets. Vet Microbiol. (2006) 115:243–9. doi: 10.1016/j.vetmic.2006.01.002, PMID: 16466864

[58] Xia B Yu J He T Liu X Su J Wang M. *Lactobacillus johnsonii* L531 ameliorates enteritis via elimination of damaged mitochondria and suppression of Sqstm1-dependent mitophagy in a *Salmonella* infantis model of piglet diarrhea. FASEB J. (2020) 34:2821–39. doi: 10.1096/fj.201901445RRR, PMID: 31908018

[59] Sterndale SO Evans DJ Mansfield JP Clarke J Sahibzada S Abraham S. Effect of Mucin 4 allele on susceptibility to experimental infection with enterotoxigenic F4 *Escherichia coli* in pigs fed experimental diets. J Anim Sci Biotechnol. (2019) 10:56. doi: 10.1186/s40104-019-0366-1, PMID: 31346463 PMC6636048

[60] Sørensen MT Vestergaard EM Jensen SK Lauridsen C Højsgaard S. Performance and Diarrhoea in piglets following weaning at seven weeks of age: challenge with *E. coli* O 149 and effect of dietary factors. Livest Sci. (2009) 123:314–21. doi: 10.1016/j.livsci.2008.12.001

[61] Amezcua MDR Friendship R Dewey C Weese S Lange Cd. THe effect of feeding fermented liquid whey + dextrose inoculated with specific lactic acid Bacteria of pig origin to weanling pigs challenged with *Escherichia coli* O149:K91:F4. Vet Ther Res Appl Vet Med (2007) 8:209–222. 17926306

[62] Lauridsen C Højberg O Kongsted H Canibe N. A Critical Review on Alternatives to Antibiotics and Pharmacological Zinc for Prevention of Diarrhoea in Pigs Post-Weaning. The Danish Veterinary and Food Administration (2017).

[63] Pedersen A. Fermenteret Vådfoder Til Smågrise. Report 510. Copenhagen, Denmark: Landsudvalget for Svin (2001).

[64] Maribo H Jensen B Hansen I Aaslyng M. Fermenteret Korn I Vådfoder Til Tungsvin. Report 547. Copenhagen, Denmark: Landsudvalget for Svin (2002).

[65] Sugiharto S Lauridsen C Jensen BB. Gastrointestinal ecosystem and immunological responses in *E. coli* challenged pigs after weaning fed liquid diets containing whey permeate fermented with different lactic acid Bacteria. Anim Feed Sci Technol. (2015) 207:278–82. doi: 10.1016/j.anifeedsci.2015.06.019

[66] Van-Winsen RL Keuzenkamp D Urlings BAP Lipman LJA Snijders JAM Verheijden JHM. Effect of fermented feed on shedding of Enterobacteriaceae by fattening pigs. Vet Microbiol. (2002) 87:267–76. doi: 10.1016/S0378-1135(02)00066-4, PMID: 12052336

[67] Hong TTT Thuy TT Passoth V Lindberg JE. Gut ecology, feed digestion and performance in weaned piglets fed liquid diets. Livest Sci. (2009) 125:232–7. doi: 10.1016/j.livsci.2009.04.013

[68] Li XQ Zhu YH Zhang HF Yue Y Cai ZX Lu QP. Risks associated with high-dose *Lactobacillus rhamnosus* in an *Escherichia coli* model of piglet Diarrhoea: intestinal microbiota and immune imbalances. PLoS One. (2012) 7:e40666. doi: 10.1371/journal.pone.0040666, PMID: 22848393 PMC3407149

[69] Pan Y Breidt F. Enumeration of viable *Listeria monocytogenes* cells by real-time PCR with propidium monoazide and ethidium monoazide in the presence of dead cells. Appl Environ Microbiol. (2007) 73:8028–31. doi: 10.1128/AEM.01198-07, PMID: 17933922 PMC2168130

[70] Spitzer F Vahjen W Pieper R Martinez-Vallespin B Zentek J. A standardised challenge model with an enterotoxigenic F4+ *Escherichia coli* strain in piglets assessing clinical traits and faecal shedding of Fae and Est-ii toxin genes. Arch Anim Nutr. (2014) 68:448–59. doi: 10.1080/1745039X.2014.968701, PMID: 25313936

[71] Rhouma M Fairbrother JM Thériault W Beaudry F Bergeron N Laurent-Lewandowski S. The fecal presence of enterotoxin and F4 genes as an Indicator of efficacy of treatment with Colistin sulfate in pigs. BMC Microbiol. (2017) 17:6. doi: 10.1186/s12866-016-0915-0, PMID: 28056796 PMC5217267

[72] Pupa P Apiwatsiri P Sirichokchatchawan W Pirarat N Nedumpun T Hampson DJ. Microencapsulated probiotic *Lactiplantibacillus plantarum* and/or *Pediococcus acidilactici* strains ameliorate Diarrhoea in piglets challenged with Enterotoxigenic *Escherichia coli*. Sci Rep. (2022) 12:7210. doi: 10.1038/s41598-022-11340-3, PMID: 35505092 PMC9065055

[73] Pomorska-Mól M Kwit K Markowska-Daniel I. Major acute phase proteins in pig serum from birth to slaughter. J Vet Res. (2012) 56:553–7. doi: 10.2478/v10213-012-0097-y

[74] Yu K Canalias F Solà-Oriol D Arroyo L Pato R Saco Y. Age-related serum biochemical reference intervals established for unweaned calves and piglets in the post-weaning period. Front Vet Sci. (2019) 6:123. doi: 10.3389/fvets.2019.00123, PMID: 31069239 PMC6491529

[75] Pié S Lallès JP Blazy F Laffitte J Sève B Oswald IP. Weaning is associated with an Upregulation of expression of inflammatory cytokines in the intestine of piglets. J Nutr. (2004) 134:641–7. doi: 10.1093/jn/134.3.641, PMID: 14988461

[76] Cao ST Wang CC Wu H Zhang QH Jiao LF Hu CH. Weaning disrupts intestinal antioxidant status, impairs intestinal barrier and mitochondrial function, and triggers Mitophagy in Piglets1. J Anim Sci. (2018) 96:1073–83. doi: 10.1093/jas/skx062, PMID: 29617867 PMC6093500

[77] Zhu LH Zhao KL Chen XL Xu JX. Impact of weaning and an antioxidant blend on intestinal barrier function and antioxidant status in pigs. J Anim Sci. (2012) 90:2581–9. doi: 10.2527/jas.2011-4444, PMID: 22896732

[78] Zwickey H Thompson B. 18 - Immune function assessment In: Pizzorno JE Murray MT, editors. Textbook of Natural Medicine. 5th ed. St. Louis (MO): Churchill Livingstone (2020). 157–65.e1.

[79] Ring C Patterson SM Bacon SL Veldhuijzen van Zanten JJCS Willemsen G Carroll D. Reliability of hematocrit during rest and stress in healthy adults. Biol Psychol. (2008) 77:63–8. doi: 10.1016/j.biopsycho.2007.09.005, PMID: 17950518

[80] Ventrella D Dondi F Barone F Serafini F Elmi A Giunti M. The biomedical piglet: establishing reference intervals for haematology and clinical chemistry parameters of two age groups with and without Iron supplementation. BMC Vet Res. (2017) 13:23. doi: 10.1186/s12917-017-0946-2, PMID: 28095847 PMC5240404

[81] Cooper CA Moraes LE Murray JD Owens SD. Hematologic and biochemical reference intervals for specific pathogen free 6-week-old Hampshire-Yorkshire crossbred pigs. J Anim Sci Biotechnol. (2014) 5:5. doi: 10.1186/2049-1891-5-5, PMID: 24410946 PMC3898366

[82] González-Ramón N Hoebe K Alava MA van Leengoed L Piñeiro M Carmona S. Pigmap/Itih4 and haptoglobin are interleukin-6-dependent acute-phase plasma proteins in porcine primary cultured hepatocytes. Eur J Biochem. (2000) 267:1878–85. doi: 10.1046/j.1432-1327.2000.01195.x, PMID: 10712621

[83] Kim K Song M Liu Y Ji P. Enterotoxigenic *Escherichia coli* infection of weaned pigs: intestinal challenges and nutritional intervention to enhance disease resistance. Front Immunol. (2022) 13:885253. doi: 10.3389/fimmu.2022.885253, PMID: 35990617 PMC9389069

[84] Li H Liu X Shang Z Qiao J. *Clostridium Butyricum* helps to alleviate inflammation in weaned piglets challenged with Enterotoxigenic *Escherichia Coli* K88. Front Vet Sci. (2021) 8:757. doi: 10.3389/fvets.2021.683863 PMC8282889 34277756

[85] Lee CY Kim SJ Park BC Han JH. Effects of dietary supplementation of bacteriophages against Enterotoxigenic *Escherichia Coli* (Etec) K88 on clinical symptoms of post-weaning pigs challenged with the Etec pathogen. J Anim Physiol Anim Nutr. (2017) 101:88–95. doi: 10.1111/jpn.12513, PMID: 27271838

[86] López-Colom P Yu K Barba-Vidal E Saco Y Martín-Orúe SM Castillejos L. I-Fabp, pig-map and Tnf-Α as biomarkers for monitoring Gut-Wall integrity in front of Salmonella Typhimurium and Etec K88 infection in a weaned piglet model. Res Vet Sci. (2019) 124:426–32. doi: 10.1016/j.rvsc.2019.05.004, PMID: 31082572

