# Supplementary material for: Liquid fermented cereals with added Pediococcus acidilactici did not reduce post-weaning diarrhea in pigs – an Escherichia coli challenge study
Source: Front Vet Sci. 2023 May 12;10:1147165. doi: 10.3389/fvets.2023.1147165 (PMC10213407; doi:10.3389/fvets.2023.1147165)
Supplement: Supplementary file 1 [file Data_Sheet_1.PDF]

Supplementary Material

1 Supplementary Figures and Tables

1.1 Supplementary Figures

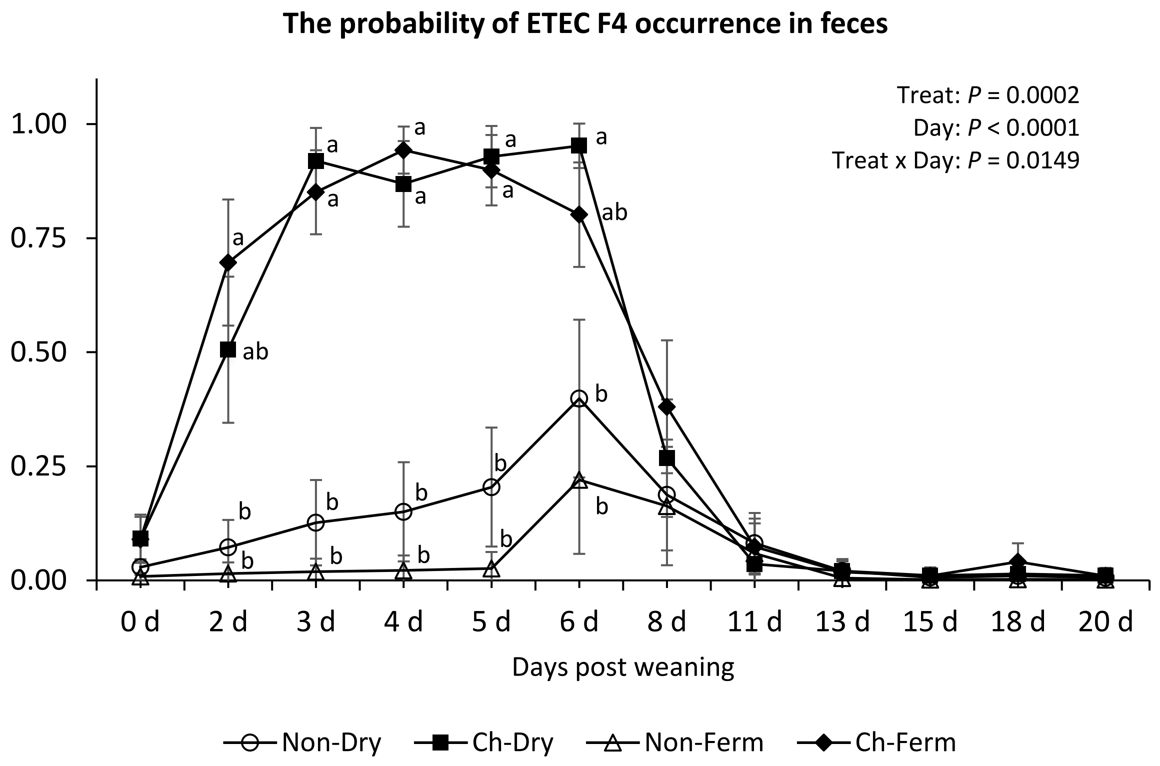

Figure S1. Effect of ETEC F4 challenge and fermented liquid feed added *P. acidilactici* on the probability of F4 occurrence in feces. The ETEC F4 was orally administered on days 1 and 2 post weaning. Data are presented as emmean ± SEM. Non-Dry: non-challenged, standard dry feed, n=8; Ch-Dry: challenged, standard dry feed, n=14; Non-Ferm: non-challenged, fermented liquid feed, n=8; Ch-Ferm: challenged, fermented liquid feed, n=16. [a,b] indicate statistical significance ($P < 0.05$).

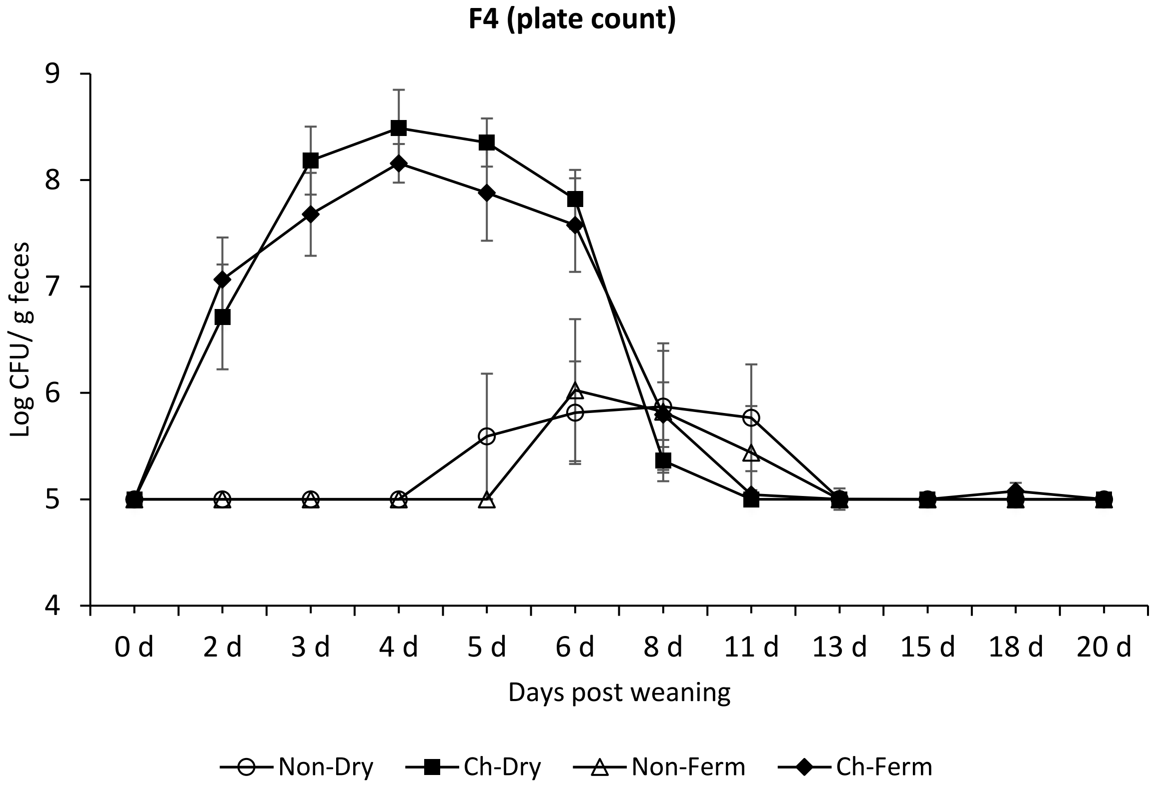

Figure S2. Effect of ETEC F4 challenge and fermented liquid feed added *P. acidilactici* on ETEC F4 (log CFU/ g feces) level in feces, the limit of detection is 5 log CFU/g feces. The ETEC F4 was orally administered on days 1 and 2 post weaning. Data are presented as mean ± SEM. Non-Dry: non-challenged, standard dry feed, n=8; Ch-Dry: challenged, standard dry feed, n=14; Non-Ferm: non-challenged, fermented liquid feed, n=8; Ch-Ferm: challenged, fermented liquid feed, n=16.

1.2 Supplementary Tables

Table S1. The ingredient composition of experiment diets: day 0 to 7 post weaning (starter diet), day 8 to 21 post weaning (weaner diet).

Ingrediens	starter diet (%)	weaner diet (%)
Wheat	50.26	52.40
Barley	23.42	21.00
Soybean meal		8.00
ViloSoy, soy protein	13.34	6.75
Potato protein, protastar	3.00	3.50
Fish meal	2.80	1.00
Palm fatty acid distillate	2.26	1.98
Sugar beet molasses	0.50	0.50
Calcium carbonate, chalk	0.76	1.25
Monocalcium phosphate	1.01	1.06
Feed salt, sodium chloride	0.31	0.36
Lysine sulphate 98	0.53	0.48
Methionine DL 98	0.12	0.11
Threonine 98	0.15	0.12
Tryptophan 99	0.05	0.04
Valine L 96.5	0.06	0.02
Vitamin premix	0.40	0.40
Ronozyme HiPhos	0.025	0.025
Chromium oxide	0.40	0.40
Luctarom Advance	0.10	0.10
Benzoic acid	0.50	0.50
TOTAL	100.00	100.00

Table S2. Effect of ETEC F4 challenge and fermented liquid feed on Hematology[1].

Item	Treatment[2]						*P*-value		
	Non-Dry	Ch-Dry	Non-Ferm	Ch-Ferm	SEM[3]	*	Treat	Day	Treat * Day
Red blood cells (10^{12} cells/L)							0.44	<0.001	0.04
Day 0	6.46	6.76	6.62	6.51	0.20				
Day 4	6.84	6.95	6.84	7.45	0.20				
Day 14	6.18	6.31	6.80	6.64	0.30				
Hemoglobin (g/L)							0.26	<0.001	0.09
Day 0	120.0	125.0	120.0	111.0	4.6	a			
Day 4	123.0	124.0	119.0	120.0	4.0	a			
Day 14	109.0	110.0	119.0	107.0	3.3	b			
Hematocrit (%)							0.68	<0.001	0.24
Day 0	37.0	38.9	37.4	34.8	1.7	b			
Day 4	41.3	41.1	40.4	41.4	1.6	a			
Day 14	34.3	34.6	36.9	34.0	1.3	b			
Reticulocytes %							0.40	<0.001	0.63
Day 0	2.81	2.93	2.08	2.61	0.70	a			
Day 4	0.87	0.93	0.79	0.61	0.20	b			
Day 14	0.83	1.21	0.87	0.95	0.30	b			
Platelets (10^9 cells/L)							0.12	0.03	0.96
Day 0	620	655	745	704	74	a			
Day 4	465	537	607	659	74	b			
Day 14	450	570	657	637	75	ab			
White blood cells (10^9 cells/L)							0.49	<0.001	0.70
Day 0	12.1	11.9	13.4	11.6	1.3	c			
Day 4	13.8	13.3	15.4	15.6	1.3	b			
Day 14	17.6	19.1	21.9	20.7	1.7	a			
Neutrophils (10^9 cells/L)							0.25	<0.001	0.61
Day 0	1.51	1.40	1.64	1.26	0.13	b			
Day 4	1.44	1.43	1.66	1.48	0.13	b			
Day 14	1.83	1.91	2.09	2.01	0.13	a			
Lymphocytes (10^9 cells/L)							0.60	<0.001	0.70
Day 0	6.74	6.98	7.11	7.13	0.67	c			
Day 4	8.06	7.61	8.77	9.06	0.66	b			
Day 14	9.89	10.95	12.11	11.46	1.00	a			
Monocytes (10^9 cells/L)							0.83	<0.001	0.96
Day 0	0.56	0.64	0.64	0.70	0.11	b			
Day 4	0.82	0.72	0.79	0.84	0.11	a			

Day 14	0.94	0.91	0.88	1.00	0.12	a			
Eosinophils (10^9 cells/L)							0.26	<0.001	<0.001
Day 0	0.08	0.12	0.07	0.09	0.02				
Day 4	0.16 ^{ab}	0.12 ^{ab}	0.23 ^{a}	0.07 ^{b}	0.04				
Day 14	0.25	0.24	0.17	0.18	0.04				

[1] Values are presented as emmeans.

[2] Non-Dry: non-challenged, standard dry feed, n=8; Ch-Dry: challenged, standard dry feed, n=14; Non-Ferm: non-challenged, fermented liquid feed, n=8; Ch-Ferm: challenged, fermented liquid feed, n=16. The ETEC F4 was orally administered on days 1 and 2 post weaning.

[3] Pooled standard error of least square means.

* For each parameter, across treatments, values within a column without a common superscript differ between days ($P < 0.05$).

[ab] Values within a row without a common superscript differ ($P < 0.05$).
